# Pharmacokinetics, pharmacodynamics, safety, and tolerability of single-dose denosumab in healthy Chinese volunteers: A randomized, single-blind, placebo-controlled study

**DOI:** 10.1371/journal.pone.0197984

**Published:** 2018-06-22

**Authors:** Qian Chen, Chaoying Hu, Yanmei Liu, Rong Song, Wenjing Zhu, Hongxin Zhao, Antonio Nino, Fan Zhang, Yun Liu

**Affiliations:** 1 Central Laboratory, Shanghai Xuhui Central Hospital and Shanghai Clinical Center, Chinese Academy of Science, Shanghai, China; 2 China Medical, GlaxoSmithKline (China) R&D Company Limited, Shanghai, China; 3 Metabolic Pathways and Cardiovascular Therapeutic Area Unit, GlaxoSmithKline, Collegeville, Pennsylvania, United States of America; 4 Clinical Pharmacology Modelling and Simulation, GlaxoSmithKline (China) R&D Company Limited, Shanghai, China; Garvan Institute of Medical Research, AUSTRALIA

## Abstract

**Background:**

Denosumab is a fully human monoclonal antibody against receptor activator of nuclear factor kappa-B ligand, a cytokine essential for the formation, function and survival of osteoclasts. This study assessed the pharmacokinetics, pharmacodynamics, safety and tolerability of single-dose denosumab (60 and 120 mg) in healthy Chinese volunteers.

**Methods:**

This randomized (3:3:2), single-blind, placebo-controlled study enrolled healthy Chinese volunteers to receive single subcutaneous injection of denosumab 60 mg, 120 mg, or placebo. Study consisted of screening period (up to 21 days), treatment and assessment period (19 weeks), and an end-of-study visit (at week 26). Denosumab pharmacokinetics and pharmacodynamics parameters were estimated using non-compartmental analysis. Safety and tolerability were assessed throughout the study.

**Results:**

A total of 63 volunteers received the study treatment and 62 (98.4%) completed the study. Denosumab serum concentrations peaked at around Day 10 with dose-proportional increase from 60 mg to 120 mg. The mean terminal half-life of denosumab 60 mg and 120 mg was 15 days and 26 days, respectively. The serum C-terminal cross-linking telopeptide of type I collagen concentration-time profiles were similar (>80% decrease within 5 days) between denosumab 60 mg and 120 mg groups. The most commonly reported adverse event (AE) was decreased blood calcium levels (denosumab 60 mg, n = 13; denosumab 120 mg, n = 13; placebo, n = 1); however only one volunteer had calcium level below the abnormality value of potential clinical importance and none of the volunteers developed symptoms of hypocalcemia. The majority of AEs were of mild to moderate intensity. There were no deaths, serious AEs, or withdrawal from study due to AEs. No clinically significant findings in vital signs or electrocardiogram were observed.

**Conclusions:**

Both denosumab 60 mg and 120 mg were well tolerated with no new safety concerns identified in healthy Chinese volunteers with similar pharmacokinetics and pharmacodynamics profiles to that of Caucasians.

**Trial registration:**

ClinicalTrial.gov NCT02135640

## Introduction

Denosumab is a fully human monoclonal antibody that selectively binds with the receptor activator of nuclear factor kappa-B ligand (RANKL), a cytokine essential for the formation, function and survival of osteoclasts.[1] By binding to RANKL on the surface of osteoclasts and their precursors denosumab inhibits osteoclast-mediated bone resorption.[2]

Denosumab is currently approved in multiple countries including European Union, United States, and Japan, but not in China.[3,4] Denosumab 60 mg is approved for the treatment of postmenopausal women with osteoporosis at high risk for fracture, to increase bone mass in men with osteoporosis at high risk for fracture, in men with prostate cancer who are at increased risk of fractures due to bone loss associated with hormone ablation.[3,5–16] Denosumab 120 mg is approved for the prevention of skeletal related events (SREs) in patients with bone metastases from solid tumors, the treatment of adults and skeletally mature adolescents with giant cell tumor of bone that is unresectable or where surgical resection is likely to result in severe morbidity.[17–20]

The pharmacokinetics (PK), pharmacodynamics (PD), safety and tolerability of denosumab 60 mg and 120 mg after single subcutaneous (SC) administration were evaluated for the first time in this Phase I study in healthy Chinese adults. Data from this study will be used to support denosumab Phase III dose determination in Chinese patients and regulatory submission in China.

## Materials and methods

### Study population

Healthy Chinese men and women aged between 18 and 65 years with body weight of at least 50 kg, body mass index (BMI) between 19 and 24 kg/m^2^ and average QT durations corrected for heart rate by Bazett’s formula (QTcB) <450 msec were enrolled in this study. Health status was determined by medical history, physical examination, laboratory tests and cardiac monitoring. Women of child-bearing potential were instructed to use appropriate contraception throughout the study and for at least 6 months after the last dose of study medication. Participation in any clinical study 30 days prior to screening, history of or current evidence of osteomyelitis or osteonecrosis of the jaw, active dental or jaw condition requiring oral surgery, non-healed dental or oral surgery or abnormal serum calcium levels were exclusionary.

The study was conducted in accordance with ICH Good Clinical Practices and applicable local regulatory requirements, principles outlined in the Declaration of Helsinki and study protocol approved by the Shanghai Xuhui Central Hospital Ethics Committee. The study details were explained to all volunteers prior to obtaining written informed consent to participation in this study.

### Study design

This randomized, single-blind (volunteer), parallel-group, placebo-controlled study was conducted in healthy Chinese volunteers between July 2014 and February 2015 at Shanghai Xuhui Central Hospital, Shanghai, China.

**Fig 1:**
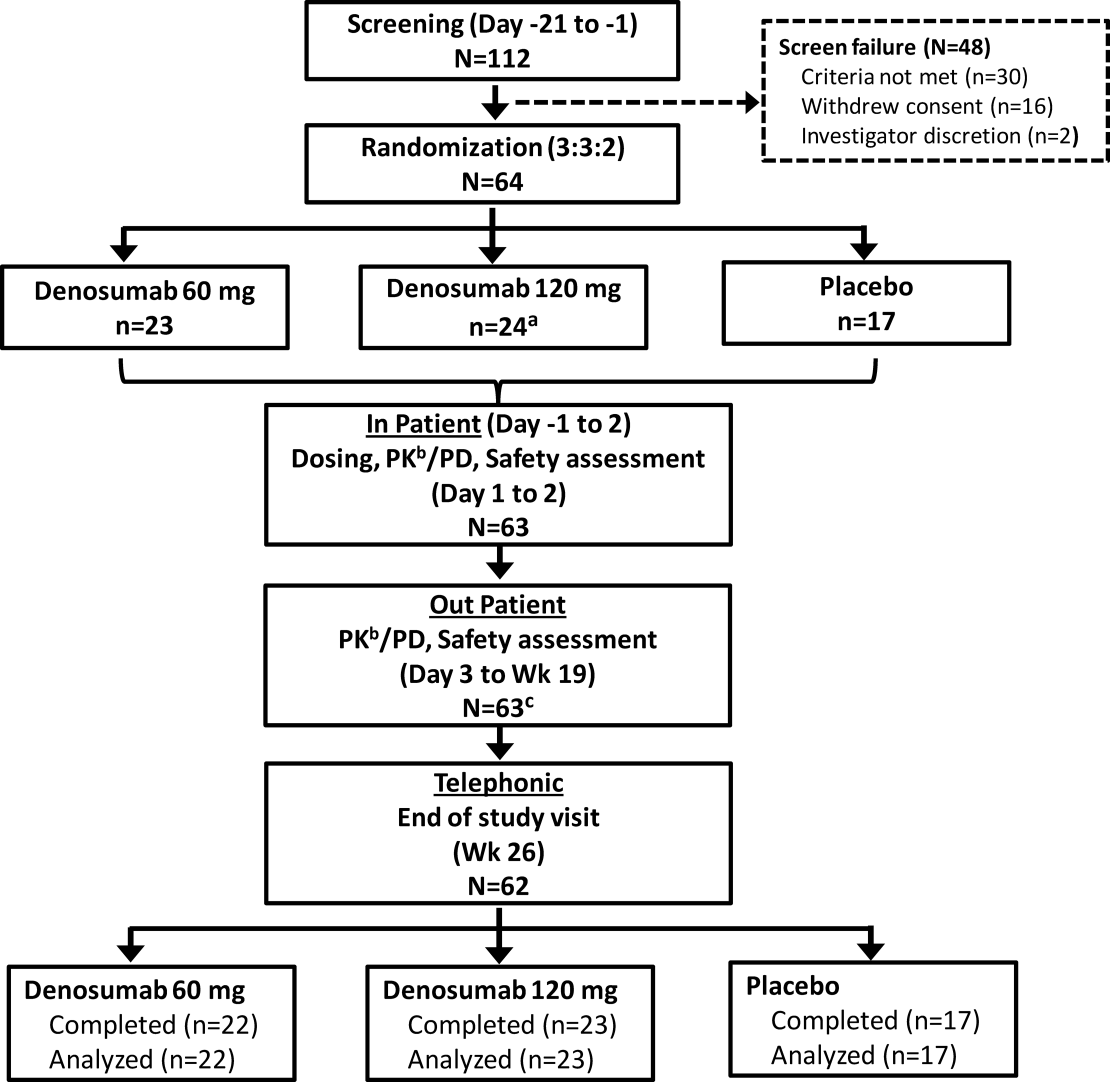
Study design and volunteers disposition. ^a^One volunteer withdrew consent before administration of study treatment. ^b^Volunteers from placebo group were not included for PK assessment. ^c^One volunteer who received denosumab 60 mg withdrew from study (investigator discretion; study day 147). PK, pharmacokinetic; PD, pharmacodynamic; Wk, week.

Volunteers were randomized (3:3:2) to one of the three treatment groups to receive SC injections of denosumab 60 mg, 120 mg, or placebo (Fig 1). The study consisted of up to 21-day screening period, a 19-week treatment and assessment period, and an end of study visit at week 26. Volunteers were hospitalized from Day -1 to Day 2. On Day 1, volunteers from denosumab 60 mg group received one injection each of denosumab 60 mg and matching placebo; volunteers from denosumab 120 mg received two injections of denosumab 60 mg (total 120 mg); and volunteers from placebo group received two matching placebo injections. Study visits and safety assessments were planned on days 3, 4, 5, 6, 8, 11 and at weeks 3, 4, 5, 7, 9, 11, 13, 15, 17, and 19. At Week 26, a follow-up assessment was conducted via telephone to identify any adverse events (AEs). All volunteers received ≥400 IU but <1000 IU/day vitamin D and ≥600 mg calcium everyday during the study. Computer-generated gender-based randomization was used, in which the single randomization schedule was split into blocks for men and women (block size 8).

### Pharmacokinetic, pharmacodynamic assessments and parameter estimation

Blood samples (3.5 mL) for PK analysis were obtained using an indwelling cannula (or by direct venipuncture) at 0 hour (pre-dose), and 1, 4, 8, and 12 hour (Day 1), Day 2, 3, 4, 5, 6, 8, 11, and at weeks 3, 4, 5, 7, 9, 11, 13, 15, 17, and 19 (post-dose). Samples were collected using serum-separating tubes and allowed to clot for 30–60 minutes and then centrifuged for 15 minutes (1500 relative centrifugal force). Supernatant serum was transferred to a 1.8 mL Nunc Cryovial and stored at -70°C before shipment to laboratory. Serum samples were analyzed for denosumab using a validated analytical method based on Enzyme-linked Immunosorbent Assay (ELISA) with the lower limit of quantification 20.0 ng/mL using human serum.

For PD analysis, samples were collected using serum-separating tubes and allowed to clot for 30–60 minutes and then centrifuged for 15 minutes (1600 relative centrifugal force). Supernatant serum (1mL) was transferred to a tube and stored at -20°C before shipment. Blood samples (3.5 mL) were obtained at Day 1 (pre-dose) and Day 2, 3, 4, 5, 6, 8, 11, and at weeks 3, 4, 5, 7, 9, 11, 13, 15, 17, and 19 (post-dose). Serum samples were assayed for serum C-terminal cross-linking telopeptide of type I collagen (CTX1), a selected PD marker, by the electrochemiluminescence immunoassay.

Denosumab PK and PD parameters were estimated using non-compartmental PK analysis in Phoenix (version 6.3, Pharsight Corporation, CA, USA). Calculations were based on the actual sampling times recorded during the study. From the plasma concentration-time data, the following PK parameters were determined: area under the concentration-time curve (AUC) from time zero (pre-dose) to last time of quantifiable concentration within volunteers across all treatments (AUC_(0-t)_), AUC from time zero to infinite time (AUC_(0-inf)_), AUC from time zero to 16 weeks (AUC_(0–16 weeks)_), maximum serum concentration (C_max_), time to reach C_max_ (T_max_), half-life (t_1/2_), apparent clearance (CL/F), apparent volume of distribution (Vd/F). From the plasma effect-time data, the following PD parameters were determined: minimum observed plasma concentration (I_min_), time to reach I_min_ (T_min_ CTX1), maximum observed percentage of inhibition (I_max_ [% Inhibition]), area under the plasma effect-time curve from time zero to last time of quantifiable concentration (AUEC_(0-t)_) and AUEC_(0–16 weeks)_.

### Safety and tolerability assessments

Safety evaluation included the monitoring of AEs and serious AEs (SAEs), clinical laboratory tests (hematology, clinical chemistry and routine urinalysis), vital signs, electrocardiograms (ECGs), and physical examinations until the Week 26 follow-up contact. All relevant information regarding an AE/SAE were recorded in the appropriate data collection tool. The severity of AEs was assessed according to the criteria of Common Terminology Criteria for AE (CTCAE_4.03).

### Statistical analysis

The sample size was determined based on study feasibility and China regulatory requirement. A total of 64 volunteers were planned to be enrolled. The safety population included volunteers who had received at least one dose of study treatment; PK and PD population included volunteers who had received at least one dose of study treatment and for whom PK and PD samples were obtained and analyzed.

All the statistical analyses were performed using the SAS software (version 9.2, SAS Institute, NC, USA). Pharmacokinetic and PD data were presented in graphical and/or tabular form and summarized descriptively and expressed using geometric mean (CVb%) except for T_max_, for which median (range) are reported. Safety data were summarized descriptively.

## Results

A total of 112 volunteers were screened and 64 volunteers were randomized to denosumab 60 mg (n = 23), denosumab 120 mg (n = 24), and placebo (n = 17) group. One volunteer withdrew consent before the administration of study treatment. A total of 63 volunteers received the study treatment, of which 62 (98.4%) completed the study and one volunteer who received denosumab 60 mg withdrew from study because of investigator discretion (Fig 1). Overall, 46 volunteers received active treatment and were included in the PK analyses. The majority (57%) of volunteers were men. Overall, the baseline and demographic characteristics including gender ratio were similar across the three treatment groups (Table 1).

**Table 1:** Demographic and baseline characteristics (safety population).

Parameter	Denosumab 60 mg (N = 23)	Denosumab 120 mg (N = 23)	Placebo (N = 17)	Total (N = 63)
Age (years), Mean (SD)	25.4 (4.2)	29.8 (7.8)	26.1 (4.6)	27.2 (6.1)
Gender, n (%)				
Men	13 (57)	13 (57)	10 (59)	36 (57)
Women	10 (43)	10 (43)	7 (41)	27 (43)
BMI (kg/m^2^), Mean (SD)	22.4 (1.2)	22.1 (1.4)	22.3 (1.3)	22.2 (1.3)
Race, n (%)				
Asian—East Asian Heritage	23 (100)	23 (100)	17 (100)	63 (100)

BMI, body mass index; SD, standard deviation.

### Pharmacokinetics and pharmacodynamics

**Fig 2:**
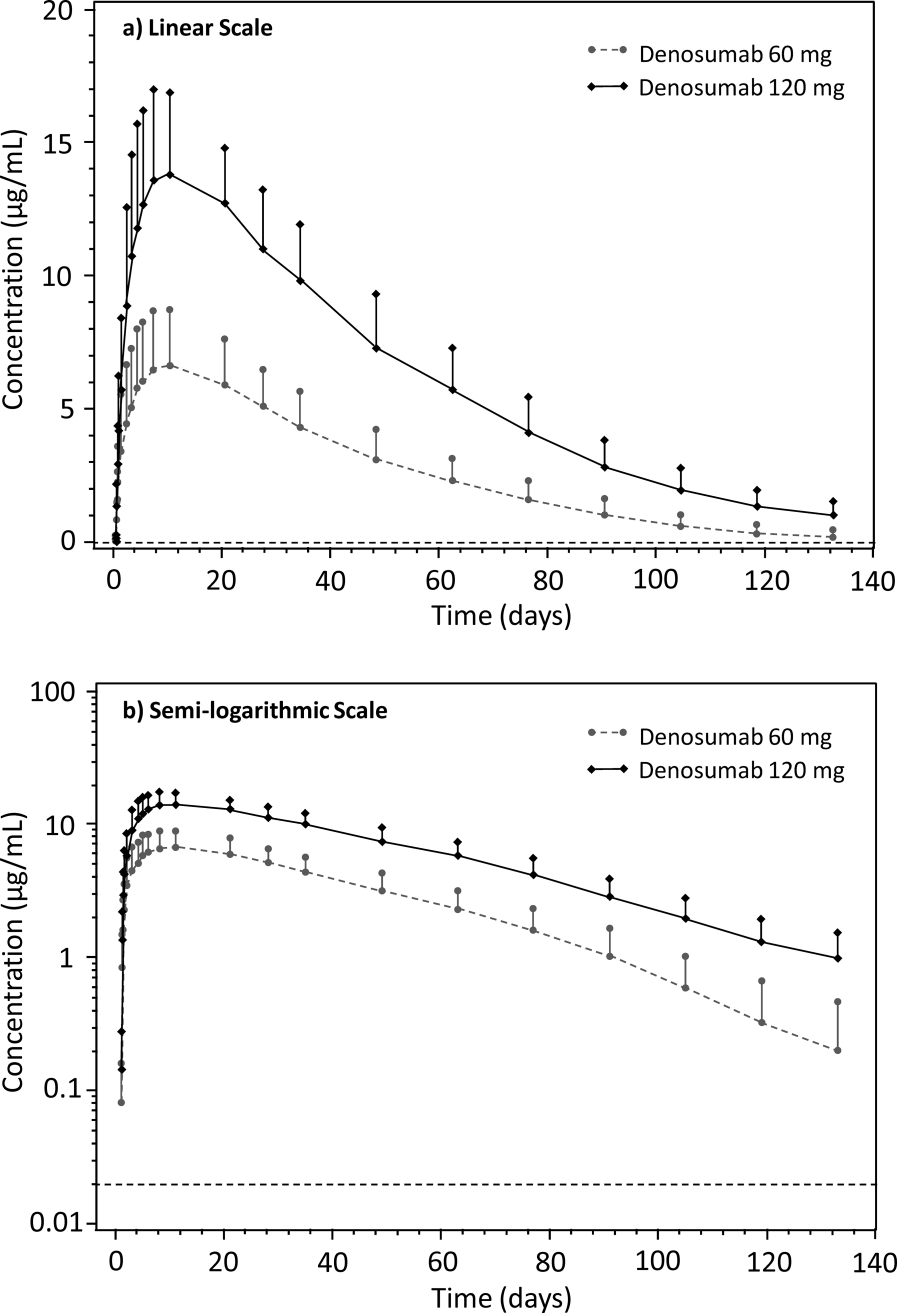
Mean serum concentration-time plot of denosumab following single subcutaneous administration at doses of 60 mg and 120 mg. Error bar represents standard deviation.

After SC administration, denosumab was absorbed slowly with peak serum concentrations observed at around Day 10 with approximate dose-proportional increase in C_max_ (2.14 fold) and AUC (2.46 fold) with increased doses from 60 mg to 120 mg. The serum concentration-time profile after C_max_ was biphasic, with an initial phase during which serum concentrations declined approximately linearly from peak followed by a more rapid terminal phase, particularly for 60 mg dose. Mean CL/F decreased from 181.7 to 147.5 mL/day for 60 mg and 120 mg group, respectively. Mean serum concentration-time profile of denosumab following single SC doses of 60 mg and 120 mg are shown in Fig 2A and 2B. The PK parameters are summarized in Table 2.

**Table 2:** Summary of pharmacokinetic parameters of denosumab after single-dose subcutaneous administration (PK population).

Parameter	Denosumab 60 mg (N = 23)	Denosumab 120 mg (N = 23)
AUC_(0-t)_ (day·μg/mL)	319.3 (37.2)	775.4 (23.2)
AUC_(0-inf)_ (day·μg/mL)	330.2 (39.3)	813.5 (25.0)
AUC_(0–16 weeks)_ (day·μg/mL)	318.7 (36.8)	752.3 (22.7)
C_max_ (μg/mL)	6.6 (34.4)	14.1 (22.0)
T_max_ (day)	10.0 (1.0–27.2)	10.0 (4.0–27.0)
t_1/2_ (day)	14.7 (57.6)	25.8 (29.1)
CL/F (mL/day)	181.7 (39.3)	147.5 (25.0)
Vd/F (mL)	3845.9 (47.3)	5495.9 (25.4)

Note: Data are presented as geometric mean (CVb%) except for T_max_, where values are median (range).

AUC_(0-t)_, area under the concentration-time curve from time zero (pre-dose) to last time of quantifiable concentration; AUC_(0-inf)_, AUC from time zero to infinity; AUC_(0–16 weeks)_, AUC from time zero to week 16; C_max_ maximum plasma concentration; CL/F, apparent oral clearance; PK, pharmacokinetic; T_max_, time to reach C_max_; t_1/2_, terminal half-life; Vd/F, apparent volume of distribution.

**Fig 3:**
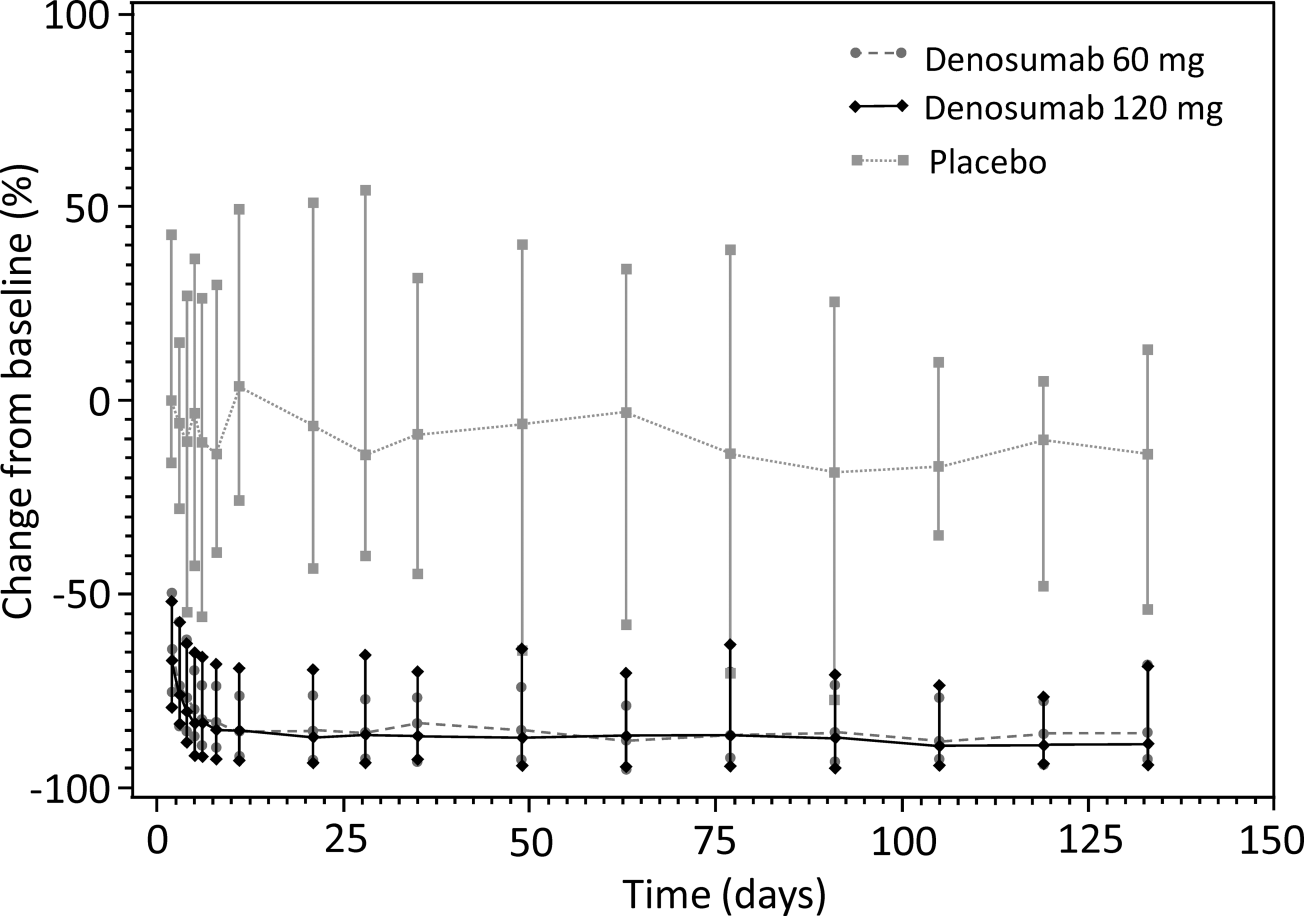
Change from baseline in median serum CTX1. Error bar represents range. CTX1, C-terminal cross-linking telopeptide of type I collagen.

The serum CTX1 concentration-time profiles were similar between denosumab 60 mg and 120 mg treatment groups. In volunteers who received denosumab, the mean serum CTX1 concentration decreased over 80% within 5 days (80.3% reduction for 60 mg group and 82% reduction for 120 mg group) after dosing and this inhibition continued over the course of the study (up to Week 19). The maximum mean decrease in serum CTX1 concentration was 89%, similar for 60 mg and 120 mg treatment groups. In the placebo group, mean serum CTX1 concentration remained near baseline over the course of the study and within the expected variance for this parameter (Fig 3; Table 3).

**Table 3:** Summary of pharmacodynamic parameters of serum CTX1 (PD population).

Parameter	Denosumab 60 mg (N = 23)	Denosumab 120 mg (N = 23)	Placebo (N = 17)
I_min_ (μg/L)	0.07 (43.1)	0.06 (41.0)	0.29 (50.4)
T_min_ (days)	52.7 (69.8)	99.3 (31.9)	41.8 (134.4)
I_max_ (% Inhibition)	88.8 (4.5)	89.2 (5.2)	40.2 (45.7)
AUEC_(0-t)_ (day*% Inhibition)	11224.0 (5.4)	11255.4 (6.9)	1528.1 (76.8)
AUEC_(0–16 weeks)_ (day*% Inhibition)	9457.6 (5.5)	9503.7 (6.9)	644.0 (406.8)

Note: Data are presented as geometric mean (CVb%).

AUEC, area under the plasma effect-time curve; CTX1, C-terminal cross-linking telopeptide of type I collagen; I_min_, minimum observed plasma concentration; I_max_, maximum observed percentage of inhibition (I_max_ [% Inhibition]); PD, pharmacodynamic; T_min_, time to reach I_min_.

### Safety and tolerability

A total of 48 volunteers (76%) experienced at least one treatment-emergent AE (TEAE) during the study. Frequency of TEAEs was comparable between denosumab 60 mg and 120 mg groups. The most frequently reported TEAE was decrease in blood calcium levels and upper respiratory tract infection. Decrease in blood calcium levels was experienced by 13 (57%) volunteers each in denosumab 60 mg and 120 mg group and by 1 (6%) volunteer in placebo group; and none of the volunteers developed symptoms of hypocalcemia (Table 4). There were no deaths or SAEs reported. None of the volunteers withdrew from the study due to AEs.

**Table 4:** Treatment-emergent adverse events occurring in ≥2 volunteers in any group (safety population).

	Denosumab 60 mg (N = 23)	Denosumab 120 mg (N = 23)	Placebo (N = 17)
**Any adverse event, n (%)**	18 (78)	19 (83)	11 (65)
Decrease in blood calcium levels	13 (57)	13 (57)	1 (6)
Increase in blood calcium levels	1 (4)	2 (9)	1 (6)
Alanine aminotransferase increased	1 (4)	2 (9)	0
Upper respiratory tract infection	8 (35)	6 (26)	3 (18)
Diarrhea	2 (9)	3 (13)	1 (6)
Abdominal pain	2 (9)	0	1 (6)
Pain in extremity	0	2 (9)	2 (12)
Contusion	0	2 (9)	0

Overall, 65% (n = 41) of volunteers experienced one or more drug-related AEs during the study (denosumab 60 mg: n = 16; denosumab 120 mg: n = 18; placebo: n = 7); the most frequently reported drug-related AE was decrease in blood calcium levels. All volunteers (n = 63) were tested for anti-denosumab antibody (ADA), however no ADA was detected in any groups at any time point (Day 1, Weeks 4, 9, and 19).

All AEs were of mild intensity except for headache in denosumab 60 mg group, and tendon rupture and nausea in placebo group which were of moderate intensity. All AEs resolved by the end of the study; except two (denosumab 60 mg group: deafness; placebo group: hemorrhoids), which were not considered related to study treatment.

Three volunteers reported abnormal clinical laboratory values of potential clinical importance (denosumab 60 mg group: elevated ALT and AST levels [n = 1]; denosumab 120 mg group: low calcium levels [n = 1]; placebo group: elevated white blood cell count [n = 1]). The elevated ALT and AST (≥2 x ULN) levels were considered by the investigator to be related to study medication, resolved at the subsequent study visit, and were not accompanied by an elevation in bilirubin. The volunteer who reported low calcium levels did not develop symptoms of hypocalcemia. At the end of the study the elevated white blood cell count had decreased but did not return to normal.

No clinically relevant findings in vital signs or ECG were observed. One woman was found to be pregnant approximately 84 days after her first dose of denosumab 120 mg; she electively terminated the pregnancy 12 days later.

## Discussion

This was the first study evaluating the PK, PD, safety, and tolerability of denosumab in Chinese population. The AEs reported in this Phase I study with single-dose (60 mg and 120 mg) of denosumab in Chinese healthy volunteers were consistent with the known safety profile of the medication, with no new safety signals.

Overall, the PK results were comparable with previous reports [21,22], suggesting no clinical relevant difference in PK profiles between Chinese and Caucasians. In previous report [21], the observed median T_max_ was 10 days (10 days in current study), mean C_max_ was 6.75 μg/mL (6.6 μg/mL in the current study) and mean AUC_0-16 weeks_ was 316 day.μg/mL (318.7 day.μg/mL in the current study) following denosumab 60 mg.[21] Denosumab CL/F was also comparable between Chinese (181.7 mL/day) and Caucasians (6.71 mL/hr [approx. 161 mL/day]).[22] The t_1/2_ was 1.7-times higher (25.4 days) than that observed in our study (14.7 days) following denosumab 60 mg (Table 5).[21,23] This may be caused by terminal sampling time point selection between studies. However, in another study the t_1/2_ following denosumab 120 mg was 28 days which is similar to our study (25.8 days).[23] In current study, dose-dependent increase (>2-fold) was observed in C_max_ and AUC in volunteers receiving 60 mg and 120 mg SC denosumab, consistent with the previous reports.[21,23]

**Table 5:** Denosumab pharmacokinetic and pharmacodynamic comparison for Chinese and non-Chinese population.

Parameter	Previous studies			Current study^a^	
	PI [21] (Denosumab 60 mg)	Denosumab [22] 1 mg/kg	Denosumab [22] 60 mg	Denosumab 60 mg	Denosumab 120 mg
Study population	-	Healthy postmenopausal women	Postmenopausal women with low BMD	Healthy volunteers	Healthy volunteers
C_max_ (μg/mL)	6.75 (1.89)	8.99 (3.34)	7.93 (2.95)	6.6 (34.4)	14.1 (22.0)
T_max_ (days), median (range)	10 (3–21)	17.5 (7–42)	26 (2.9–32)	10.0 (1.0–27.2)	10.0 (4.0–27.0)
AUC_(0–16 weeks)_ (μg·day/mL)	316 (101)	538 (224)^b^	503 (239)^c^	318.7 (36.8)	752.3 (22.7)
t_1/2_ (days)	25.4 (8.5)	30.2 (7.04)	25.4 (8.47)	14.7 (57.6)	25.8 (29.1)
CL/F	-	6.61 (2.93) (mL/hr)	6.71 (5.00) (mL/hr)	181.7 (39.3) (mL/day)	147.5 (25.0) (mL/day)

Note: Data are presented as mean (SD) except T_max_.

^a^geometric mean (CVb%) except for T_max_ for current study

^b^AUC_(0-inf)_; ^c^AUC_(0-tau)_.

AUC_(0–16 weeks)_, area under the concentration-time curve from time zero to week 16; BMD, bone mineral density; C_max_ maximum plasma concentration; CL/F, apparent oral clearance; PI, prescribing information; SD, standard deviation; T_max_, time to reach C_max_; t_1/2_, terminal half-life.

The CTX1 is a specific marker of bone reabsorption. Serum CTX1 levels are proportional and indicative of osteoclastic activity at the time of blood sampling. In our study, more than 80% reduction in serum CTX1 concentration were observed in both denosumab 60 mg and 120 mg groups from Day 5 to Week 19 following single SC administration of denosumab. In Caucasians, CTX1 level reduced to similar extent by approximately 85% after 3 days of administration of single SC denosumab 60 mg dose.[21] Maximal reduction of serum CTX1 (89%) was achieved in the current study, similar as the >87% maximum of CTX1 inhibition achieved in Caucasians.[21] Denosumab was well tolerated in this study with no new safety concerns. There were no deaths, SAEs or discontinuations due to AE during the study. Most of the TEAEs were of mild intensity. Overall, the TEAEs were similar between the two denosumab treatment groups. The most frequently reported TEAEs among treatment groups were decreased blood calcium, upper respiratory tract infection, diarrhea, and pain in extremity. These events were also observed in previous studies, however with varying frequency.[6,9,10,13,14,24–27] Among volunteers with decreased serum calcium, none of them reported clinical manifestations of hypocalcemia. In previous studies, back pain, pain in extremity, hypercholesterolemia, musculoskeletal pain, cystitis, arthralgia, and nasopharyngitis were the most commonly reported AEs.

The study that assessed the clinical use of denosumab in patients with post-menopausal osteoporosis (NCT02014467) among Chinese population was completed recently and will be reported later; however another study assessing the usefulness of denosumab in patients with bone metastases from solid tumors (NCT01920568) among Chinese population is ongoing.

In conclusion, this study demonstrated that denosumab treatment was well tolerated with no new safety concerns in healthy Chinese volunteers. Single SC dose of denosumab 60 mg and 120 mg demonstrated similar PK and PD profiles in Chinese and that of in Caucasians which supports the dose selection of denosumab in Chinese patients.

## Supporting information

S1 CONSORT Checklist CONSORT checklist. (DOC)

S1 File Protocol redacted_ A randomized, single-blind, parallel-group, placebo-controlled, single-dose study to evaluate the safety, tolerability, pharmacokinetics, and pharmacodynamics of denosumab administered subcutaneously to healthy adults in China. (PDF)
