# Supplementary material for: Pharmacokinetics, pharmacodynamics, safety, and tolerability of single-dose denosumab in healthy Chinese volunteers: A randomized, single-blind, placebo-controlled study
Source: PLoS One. 2018 Jun 22;13(6):e0197984. doi: 10.1371/journal.pone.0197984 (PMC6014657; doi:10.1371/journal.pone.0197984)
Supplement: S1 File — (PDF) [file pone.0197984.s002.pdf]

TITLE PAGE

Division: Worldwide Development
Retention Category: GRS019
Information Type: Protocol Amendment

**Title:**	A randomized, single-blind, parallel-group, placebo-controlled, single-dose study to evaluate the safety, tolerability, pharmacokinetics, and pharmacodynamics of denosumab administered subcutaneously to healthy adults in China

Compound Number: GSK2371746

Effective Date: 01-MAY-2014

Protocol Amendment Number: 2

Description:

Denosumab is currently under development for the treatment of bone loss indications. The primary purpose of the study is to assess the safety and tolerability of denosumab in healthy adult Chinese volunteers. The study will also characterize the pharmacokinetic and pharmacodynamic properties of denosumab after single, subcutaneous administration.

Subject: Osteoporosis, Safety, Tolerability, Pharmacokinetics, Pharmacodynamics

Author:

[redacted] GCSP

[redacted] CPSSO

[redacted] Discovery Biometrics

[redacted] MPC TA Unit

[redacted] CPMS

[redacted] CPSSO

[redacted] CTS

[redacted] MPC TA Unit

[redacted] CPSSO

Copyright 2014 the GlaxoSmithKline group of companies. All rights reserved. Unauthorised copying or use of this information is prohibited.

Revision Chronology:

RM2010/00059/00	2010-MAY-27	Original
RM2010/00059/01	2013-NOV-07	Amendment No.01:

Change the study subjects from "healthy adults in Hong Kong and Taiwan" to "healthy adults in China".

Update the document for [redacted] and [redacted] in authors list.

Update the unit to [redacted] as sponsor on the signatory page.

Change responsible person on the sponsor/ medical monitor information page.

Change sponsor registered address.

Provide agency indentifying number.

Delete "SGOT" and "SGPT" in the abbreviations.

Modify trademark information.

Update information of clinical studies in Section 1.1.3.1 Safety profile.

Replace the updated edition of Clinical Investigator's Brochure as reference.

Simplify study rationale.

Change the number of subjects from 128 to 64.

Clarify ±2 days for Visit Window from Week 3 to follow-up in Time and Events Table.

Change the age range from "between 20 and 65" to "between 18 and 65".

RM2010/00059/02	2014-MAY-01	Amendment No.: 2
		Clarify the description of hypersensitivity in the safety profile.<br><br>Clarify denosumab 60 mg and 120 mg are applied for approval in China.<br><br>Add information of atypical femoral fracture and hypersensitivity in the risk management assessment.<br><br>Revise the dose of calcium supplementation from "at least 1000 mg" to "at least 600 mg".<br><br>Update time and events table: specify the Day and Week, specify the items of early withdrawal, add chest X- ray exam and syphilis test at screening, clarify urine pregnancy test will be used for women in Day -1, add urine pregnancy test for women during the study period, add physical examinations, ECG and clinical chemistry, hematology and urinalysis on Day -1.<br><br>Modify the exclusion criteria to exclude subjects who are positive for syphilis.<br><br>Clarify the serum hCG test at screening or urine hCG test on day -1 to exclude pregnant females.<br><br>Modify the exclusion criteria to exclude subjects with clinical significant abnormalities through chest radiograph.<br><br>Delete redundancy description of certain diseases history in exclusion criteria.<br><br>Specify the collection of blood sample in study assessments and procedures.<br><br>Shorten the interval between the examination of vital signs and ECG from 10 minutes to 5 minutes.

Modify the laboratory information of PK sample detection and analysis.

Specify the volume of blood sampling and detecting method of s-CTX1.

Specify the volume of blood sampling and detecting methods of denosumab antibody.

Modify the requirement of collection screen failure information.

Clarify that the single-blind is blinded to subjects only, and delete the remnant description of unblinding.

Add atypical femoral fracture into adverse events and serious adverse events.

Add information about atypical femoral fracture adjudication committee.

SPONSOR SIGNATORY

[redacted] 1 May 2014

Date

Medicine Development Leader

SPONSOR/MEDICAL MONITOR INFORMATION PAGE

Medical Monitor and Sponsor Contact Information:

Role	Name	Day Time Phone Number	After-hours Phone/Cell/ Pager Number	Fax Number	GSK Address
Primary Medical Monitor	[redacted] MD	[redacted]	[redacted]	[redacted]	No.1 building, 917 Halei Road<br>Zhangjiang Hi-tech Park, Pudong, shanghai
Secondary Medical Monitor	[redacted] MD	[redacted]	[redacted]	[redacted]	No.1 building, 917 Halei Road<br>Zhangjiang Hi-tech Park, Pudong, shanghai
SAE fax number				[redacted]	

Sponsor Registered Address:

GlaxoSmithKline
2301 Renaissance Boulevard
Building #510
Post Office Box 61540
King of Prussia, Pennsylvania 19406
Telephone number: [redacted]

Regulatory Agency Identifying Number(s): 2013L02177

INVESTIGATOR PROTOCOL AGREEMENT PAGE

I confirm agreement to conduct the study in compliance with the protocol, as amended by this protocol amendment.

Investigator Name:		
Investigator Address:		
Investigator Phone Number:		
Investigator Signature		Date

TABLE OF CONTENTS

PAGE

ABBREVIATIONS 11

1. INTRODUCTION 15
   1.1. Background 15
      1.1.1. Bone Disease and RANKL 15
      1.1.2. Denosumab Background 15
      1.1.3. Clinical Experience 16
         1.1.3.1. Safety Profile 16
      1.1.4. Pharmacodynamic Profile in Phase 1 Studies 18
      1.1.5. Pharmacokinetic Profile 18
   1.2. Study Rationale 19
   1.3. Dose Rationale 19
   1.4. Summary of Risk Management 19

2. OBJECTIVE(S) 22
   2.1. Primary 22
   2.2. Secondary 22

3. ENDPOINT(S) 22
   3.1. Primary 22
   3.2. Secondary 22

4. INVESTIGATIONAL PLAN 22
   4.1. Study Design/Schematic 22
      4.1.1. Screening 23
      4.1.2. Treatment 23
   4.2. Discussion of Design 24
   4.3. Treatment Assignment 24
   4.4. Investigational Product Dosage/Administration 24
      4.4.1. Dose Adjustment/Stopping Safety Criteria 24
   4.5. Time and Events Table 26

5. STUDY POPULATION 28
   5.1. Number of Subjects 28
   5.2. Eligibility Criteria 28
      5.2.1. Inclusion Criteria 28
      5.2.2. Exclusion Criteria 29
      5.2.3. Other Eligibility Criteria Considerations 30

6. DATA ANALYSIS AND STATISTICAL CONSIDERATIONS 30
   6.1. Hypotheses and Treatment Comparisons 30
   6.2. Sample Size Considerations 31
      6.2.1. Sample Size Assumptions 31
      6.2.2. Sample Size Sensitivity 31
      6.2.3. Sample Size Re-estimation 31
   6.3. Data Analysis Considerations 31
      6.3.1. Interim Analysis 31
      6.3.2. Final Analyses 31
         6.3.2.1. Safety Analyses 31

6.3.2.2. Pharmacokinetic Analyses 31
6.3.2.3. Pharmacokinetic/Pharmacodynamic Analyses 32
6.3.2.4. Pharmacodynamic/Biomarker Analyses 32
6.3.2.5. Efficacy Analyses 33

7. STUDY ASSESSMENTS AND PROCEDURES 33
7.1. Demographic/Medical History Assessments 33
7.2. Safety 34
7.2.1. Physical Examinations 34
7.2.2. Vital Signs 34
7.2.3. Height 34
7.2.4. Weight 34
7.2.5. Body Mass Index 35
7.2.6. Electrocardiogram (ECG) 35
7.2.7. Clinical Laboratory Assessments 35
7.3. Pregnancy 36
7.3.1. Time period for collecting pregnancy information 36
7.3.2. Action to be taken if pregnancy occurs 36
7.3.3. Action to be taken if pregnancy occurs in a female partner of a male study subject 36
7.4. Pharmacokinetics 36
7.4.1. Blood Sample Collection 36
7.4.2. Sample Analysis 37
7.5. Biomarker(s)/Pharmacodynamic Markers 37
7.5.1. Confirmed Biomarkers/Pharmacodynamic Markers 37
7.6. Denosumab Antibody (Seroreactivity) Assay 37

8. LIFESTYLE AND/OR DIETARY RESTRICTIONS 37
8.1. Contraception Requirements 37
8.1.1. Female Subjects 37
8.2. Meals and Dietary Restrictions 38
8.3. Caffeine, Alcohol, and Tobacco 38
8.4. Activity 39

9. CONCOMITANT MEDICATIONS AND NON-DRUG THERAPIES 39
9.1. Permitted Medications 39
9.2. Prohibited Medications 39
9.3. Non-Drug Therapies 39

10. COMPLETION OR EARLY WITHDRAWAL OF SUBJECTS 40
10.1. Subject Completion 40
10.2. Subject Withdrawal Criteria 40
10.3. Subject Withdrawal Procedures 40
10.3.1. Subject Withdrawal from Study 40
10.4. Treatment After the End of the Study 40
10.5. Screen and Baseline Failures 40

11. INVESTIGATIONAL PRODUCT(S) 41
11.1. Description of Investigational Product 41
11.2. Handling and Storage 41
11.3. Blinding 41
11.4. Packaging and Labeling 41

11.5. Preparation/Handling/Storage/Accountability 41
11.6. Assessment of Compliance 42
11.7. Treatment of Investigational Product Overdose 42

12. ADVERSE EVENTS (AE) AND SERIOUS ADVERSE EVENTS (SAE) 43
12.1. Definition of Adverse Events 43
12.2. Definition of Serious Adverse Events 44
12.3. Oral Adverse Events 45
12.4. Atypical Femoral Fracture 45
12.5. Method of Detecting AEs and SAEs 46
12.6. Recording of AEs and SAEs 46
12.7. Evaluating AEs and SAEs 46
12.7.1. Assessment of Intensity 46
12.7.2. Assessment of Causality 47
12.8. Follow-up of AEs and SAEs 47
12.9. Prompt Reporting of SAEs to GSK 48
12.10. Regulatory Reporting Requirements For SAEs 48

13. LIVER CHEMISTRY FOLLOW-UP PROCEDURES 49

14. STUDY CONDUCT CONSIDERATIONS 50
14.1. Posting of Information on Clinicaltrials.gov 50
14.2. Regulatory and Ethical Considerations, Including the Informed Consent Process 50
14.2.1. Urgent Safety Measures 50
14.3. Quality Control (Study Monitoring) 50
14.4. Quality Assurance 51
14.5. Study and Site Closure 51
14.6. Records Retention 51
14.7. Provision of Study Results to Investigators, Posting to the Clinical Trials Register and Publication 52
14.8. Data Management 52
14.9. Osteonecrosis of the Jaw Adjudication Committee 53
14.10. Atypical Femoral Fracture Adjudication Committee (AFFAC) 53

15. REFERENCES 54

APPENDICES 56
Appendix 1: Protocol Amendment Changes 56

ABBREVIATIONS

ADT	Androgen deprivation therapy
AE	Adverse Event
AFF	Atypical Femoral Fracture
AFFAC	Atypical Femoral Fracture Adjudication Committee
AIT	Aromatase inhibitor therapy
ALT	Alanine aminotransferase
AST	Aspartate aminotransferase
AUC	Area under concentration-time curve
AUC(0-∞)	Area under the concentration-time curve from time zero (pre-dose) extrapolated to infinite time
AUC(0-x)	Area under the concentration-time curve from zero (pre-dose) to some fixed nominal time x
AUC(0-t)	Area under the concentration-time curve from time zero (pre-dose) to last time of quantifiable concentration within a subject across all treatments
AUC(0-τ)	Area under the concentration-time curve over the dosing interval
AUEC	Area under the plasma effect-time curve
β-HCG	Beta-human chorionic gonadotropin
BA	Bioavailability
BE	Bioequivalence
BMD	Bone mineral density
BMI	Body mass index
BMU	Basic multicellular units
BP	Blood pressure
BPM	Beat Per Minute
BQL	Below the quantification limit
BUN	Blood urea nitrogen
CBC	Complete blood count
CCDP	Complete Clinical Development Plan
CI	Confidence Interval
CIB	Clinical Investigator's Brochure
CLr	Renal clearance
CL	Systemic clearance of parent drug
CL/F	Apparent clearance following oral dosing
Cmax	Maximum observed concentration
Cmin	Minimum observed concentration
Cτ	Pre-dose (trough) concentration at the end of the dosing interval
Ct	Last observed quantifiable concentration
CO_2	Carbon dioxide
CPK	Creatine phosphokinase
CPMS	Clinical Pharmacokinetics Modelling & Simulation
CPSR	Clinical Pharmacology Study Report
Cr	Creatinine
CRF	Case Report Form

CRO	Contract Research Organization
CPSSO	Clinical Pharmacology Science and Study Operations
CV	Coefficient of variance
DB	Discovery Biometrics
DBP	Diastolic blood pressure
DMPK	Drug Metabolism and Pharmacokinetics
DNA	Deoxyribonucleic acid
ECG	Electrocardiogram
EDC	Electronic data capture
EISR	Expedited Investigator Safety Report
FDA	Food and Drug Administration
FSH	Follicle Stimulating Hormone
FTIH	First time in humans
GCP	Good Clinical Practice
GCSP	Global Clinical Safety and Pharmacovigilance
GGT	Gamma glutamyltransferase
GLP	Good Laboratory Practice
GLS	Geometric Least-Squares
GSK	GlaxoSmithKline
HBsAg	Hepatitis B surface antigen
hCG	Human chorionic gonadotropin
HIV	Human Immunodeficiency Virus
h/hr	Hour(s)
HR	Heart rate
IB	Investigator's Brochure
ICH	International Conference on Harmonization of Technical Requirements for Registration of Pharmaceuticals for Human Use
IDSL	Integrated Data Standards Library
IEC	Independent Ethics Committee
IND	Investigational New Drug
IP	Investigational Product
IRB	Institutional Review Board
IU	International Unit
IV	Intravenous
Kg	Kilogram
L	Liter
LFTs	Liver function tests
ln	Naperian (natural) logarithm
LOQ	Limit of quantification
LLQ	Lower limit of quantification
µg	Microgram
µL	Microliter
MCH	Mean corpuscular hemoglobin
MCHC	Mean corpuscular hemoglobin concentration
MCV	Mean corpuscular volume
MedDRA	Medical Dictionary for Regulatory Activities

Mg	Milligrams
mL	Milliliter
MSDS	Material Safety Data Sheet
msec	Milliseconds
NQ	Non-quantifiable concentration measured as below LLQ
ONJ	Osteonecrosis of the Jaw
ONJAC	Osteonecrosis of the Jaw Adjudication Committee
PD	Pharmacodynamic
PGx	Pharmacogenetics
PK	Pharmacokinetic
PMO	Postmenopausal osteoporosis
PSRI	Periodic Safety Reports for Investigators
QC	Quality control
QD	Once daily
QTcB	QT duration corrected for heart rate by Bazett's formula
QTcF	QT duration corrected for heart rate by Fridericia's formula
RANKL	RANK Ligand
RAP	Reporting and Analysis Plan
RBA	Relative Bioavailability
RBC	Red blood cells
SAE	Serious adverse event(s)
SAS	Statistical Analysis Software
s-BALP	Serum bone-specific alkaline phosphatase
SC	Subcutaneous
s-CTX1	Serum carboxyterminal cross-linking telopeptide of Type I collagen
SD	Standard deviation
SOP	Standard Operating Procedure
SPM	Study Procedures Manual
SUSAR	Suspected, Unexpected, Serious Adverse drug Reaction
TNF	Tumor necrosis factor
t	Time of last observed quantifiable concentration
t½	Terminal phase half-life
τ	Dosing interval
tlag	Lag time before observation of drug concentrations in sampled matrix
tlast	Time of last quantifiable concentration
tmax	Time of occurrence of Cmax
u-NTX	Urinary amino-terminal cross-linking telopeptide of type I collagen
ULN	Upper limit of normal
UK	United Kingdom
US	United States
Vd/F	Apparent volume of distribution after extravascular (e.g., oral) administration
WBC	White blood cells

Trademark Information

Trademarks of the GlaxoSmithKline group of companies
NONE

Trademarks not owned by the GlaxoSmithKline group of companies
Amgen
Chiron RIBA
InForm
PPD
SAS
WinNonlin

1. INTRODUCTION

1.1. Background

1.1.1. Bone Disease and RANKL

Bone remodeling is characterized by two activities, resorption of old bone by osteoclasts and formation of new bone by osteoblasts. These processes are coupled temporally and spatially by recruitment of teams of osteoclasts and osteoblasts (basic multicellular units or BMUs) that work in concert to remove and replace packets of bone. Bone remodeling is measured using the bone turnover rate and depends on the activation frequency (birth rate of new BMUs), level of activity of osteoclasts and osteoblasts, and termination rate of BMUs [Simonet, 1997].

Perturbations in the balance between bone formation and resorption can lead to generalized osteoporosis (estrogen deficiency and aging) or local bone lysis (resulting from rheumatoid arthritis, lytic bone metastases, and osteomyelitis) [Burr, 1989].

RANK ligand (RANKL; a member of the tumor necrosis factor [TNF] family of proteins) was originally identified in activated T-cells [Anderson, 1997]. RANKL has been well documented as an essential factor in the formation, activation, and survival of osteoclasts [Burgess, 1999; Lacey, 1998; Yasuda, 1998], which is the sole cell type responsible for bone resorption. RANKL production is increased when estrogen is decreased (in menopause and in conditions of hormone ablation) which leads to an increase in bone resorption, and excessive RANKL has been implicated in bone diseases associated with increased bone resorption [Eghbali-Fatourechi, 2003; Kostenuik, 2001; Bucay, 1998]. Knockout mice lacking RANKL demonstrate absence of osteoclasts and increased bone density [Kong, 1999] and preclinical models have demonstrated that inhibiting RANKL leads to significant improvements in cortical and trabecular bone density, volume, and strength.

1.1.2. Denosumab Background

Denosumab is a fully human monoclonal IgG_2 antibody to RANKL that binds with high affinity (Kd 3×10^{-12} M) and specificity to the soluble and cell membrane-bound forms of human RANKL. Denosumab is highly specific because it binds only to RANKL and not to other members of the TNF family, including TNFα, TNFβ, TNF-related apoptosis-inducing ligand, or CD40 ligand [Elliott, 2006]. Denosumab binding to RANKL prevents RANK activation and inhibits the formation, activation, and survival of osteoclasts. As a consequence, bone resorption and cancer-induced bone destruction is reduced. Denosumab is being investigated as a therapeutic agent in all bone diseases characterized by excessive bone resorption, such as primary and secondary osteoporosis, metastatic bone diseases, and other diseases involving bone loss associated with increases in osteoclast function.

1.1.3. Clinical Experience

The safety and efficacy of denosumab were evaluated for recently submitted marketing applications for bone loss indications. Safety and efficacy were primarily evaluated from four Phase 3 studies of denosumab (60 mg 6-monthly (Q6M)) in postmenopausal women with osteoporosis or low bone mass, bone loss associated with androgen deprivation therapy (ADT) for prostate cancer or bone loss associated with aromatase inhibitor therapy (AIT) for breast cancer. Summaries of key safety and efficacy results are provided below.

1.1.3.1. Safety Profile

Safety and tolerability of denosumab has been evaluated in a series of placebo-controlled, Phase 1 studies in healthy, adult male and female volunteers. These studies included single- and multiple-dose regimens in healthy postmenopausal women and men ≥ 50 years of age. Two additional Phase 1 studies evaluated denosumab in subjects with cancer-related bone metastases.

A comprehensive clinical program is currently evaluating denosumab as a potential treatment for postmenopausal osteoporosis (PMO), male osteoporosis, bone loss due to hormone-ablation therapy in subjects with cancer, inhibition of structural damage in subjects with rheumatoid arthritis, prevention of bone metastases in at risk populations, prevention of skeletal-related events in subjects with advanced malignancies involving bone (including multiple myeloma), treatment of multiple myeloma, treatment of giant cell tumor, and treatment of hypercalcemia of malignancy. As of May 2013, approximately 32,800 subjects have enrolled in clinical studies and have received at least 1 dose of investigational product (i.e., denosumab, matching placebo, or active control). Cumulative doses up to 1080 mg over 6 months have been evaluated in the advanced cancer setting without evidence of dose-limiting toxicity. Repeated fixed subcutaneous (SC) doses of up to 210 mg Q6M have been studied for up to 24 months in postmenopausal women with low bone mineral density (BMD). Additionally, SC doses of up to 180 mg 4- or 12-weekly (Q4W or Q12W) have been studied for up to 26 weeks in subjects with cancer-related bone metastases, and SC doses of 60 and 180 mg Q6M have been studied for up to 12 months in subjects with rheumatoid arthritis. Key safety findings from these studies of denosumab support the following conclusions:

- Denosumab administration was generally well-tolerated; adverse events were predominantly mild to moderate in severity and of similar incidence overall between denosumab and placebo groups.
  - The overall incidences of adverse events, serious adverse events, and adverse events leading to treatment withdrawal were generally similar between denosumab and placebo groups.
  - Few subjects (< 1%) permanently discontinued denosumab treatment due to treatment-related adverse events over the 2- to 3-year duration of the 4 key Phase 3 studies.
- In the completed Phase 3 protocols, denosumab administration was associated with mild (i.e., median serum calcium decreases from baseline ≤ 3%), transient decreases

in serum calcium, which had no apparent clinical significance. Decreases in serum calcium to ≤ 7.5 mg/dL occurred with 0.04% incidence in both treatment groups (calcium and vitamin D supplementation was provided as standard-of-care in Phase 3 denosumab study protocols). In the Oncology development program, an increase in the incidence of hypocalcemia was observed (9.3% for denosumab; 4.7% Zometa), necessitating the monitoring and correction of serum calcium prior to the first dose of denosumab in the Oncology program, only.

- Extensive evaluations of cardiovascular data, including electrocardiograms (ECGs), cardiovascular adverse events and serious adverse events, external adjudication of all cardiovascular serious adverse events, and an aortic calcification substudy using lateral spine x-rays revealed no evidence of cardiovascular risk with denosumab administration.
- Subject incidences of infection adverse events (non-serious and serious events combined) were generally balanced between the treatment groups; a somewhat greater incidence of skin infections, predominantly cases of cellulitis reported as serious adverse events was observed in PMO patients.
- Eczema adverse events were more frequent in denosumab-treated postmenopausal women relative to placebo.
  - Among subjects with postmenopausal bone loss, eczema adverse events occurred in 3.1% of denosumab-treated subjects compared with 1.7% of placebo-treated subjects; the incidence of these events was balanced among subjects with bone loss due to androgen-deprivation or aromatase therapy (1.2% denosumab; 1.4% placebo).
- Subject incidences of malignancy adverse events were generally balanced between treatment groups.
- No adverse effects of denosumab on bone safety were observed:
  - Fracture healing complications were infrequent, and the subject incidence of such events was balanced between treatment groups.
  - Osteonecrosis of the jaw (ONJ) has been observed in patients receiving denosumab for advanced cancer, but rarely in patients with PMO.
  - Bone histology was normal following treatment with denosumab, either in treatment-naïve subjects or in subjects previously treated with alendronate.
- Denosumab did not result in increased incidence of hypersensitivity reactions or potential clinical sequelae of hypersensitivity in clinical trials. In the post-marketing setting, rare events of drug-related hypersensitivity, including anaphylaxis have been reported in patients receiving denosumab. Denosumab is contraindicated in individuals with clinically significant hypersensitivity.

With regard to Clinical Immunology findings, comprehensive evaluations indicated that denosumab posed little risk for immunogenicity. Samples from > 18000 denosumab-treated subjects (i.e., from all studies included in the marketing application) indicated that binding antidenosumab antibodies were observed in < 1% of subjects after administration of denosumab. With follow-up testing of these subjects, the antidenosumab antibodies

typically did not persist. There was no evidence of altered safety or efficacy profiles associated with antibodies in these subjects. To date, no denosumab-treated subject has tested positive for neutralizing antibodies in a cell-based bioassay.

1.1.4. Pharmacodynamic Profile in Phase 1 Studies

Overall, the pharmacodynamic profile of denosumab appeared similar across all of the subject populations studied, which included healthy postmenopausal women (including Japanese population), healthy men ≥ 50 years of age, subjects with advanced cancer and bone metastases (breast cancer [including a study population of Japanese women], other solid tumors [excluding lung], and multiple myeloma), and subjects with rheumatoid arthritis. The results from these studies indicated that SC administration of 60 mg denosumab caused a rapid reduction in bone resorption within 6 hours, as assessed by the serum marker carboxy-terminal cross-linking telopeptide of Type I collagen (s-CTX1) (approximately 70% reduction), with an approximately 85% reduction occurring by 3 days. Serum CTX1 reductions were maintained for 6 months after the 60-mg dose, and partially attenuating from a maximal reduction of ≥ 87% to reductions approximately 45% or greater (range 45% to 80%), reflecting the reversibility of denosumab's effects on bone remodeling. These effects were sustained with continued treatment.

Decreases in mean serum bone-specific alkaline phosphatase (s-BALP) levels were observed after denosumab administration but occurred later than changes in urinary amino-terminal cross-linking telopeptide of type I collagen (u-NTX)/urinary creatinine (uCr) and serum CTX1. These results are consistent with denosumab acting primarily as a bone antiresorptive agent and demonstrated that coupling between bone resorption and formation remained intact.

1.1.5. Pharmacokinetic Profile

The pharmacokinetics of denosumab following intravenous (IV) or SC administration has been studied at doses up to 3 mg/kg or 210 mg in various populations, including healthy postmenopausal women (including a study population of Japanese women), healthy men ≥ 50 years of age, postmenopausal women with low bone density, subjects with advanced cancer with bone metastases (breast cancer [including a study population of Japanese women], other solid tumors [excluding lung], giant cell tumor, and multiple myeloma), and subjects with rheumatoid arthritis.

Following SC administration, denosumab exhibited dose dependent, nonlinear pharmacokinetics over a wide range (as observed for other monoclonal antibodies). However, approximately dose-proportional increases in exposure were observed for doses ≥ 60 mg, consistent with saturable and nonsaturable mechanisms of elimination. For a 60-mg or 120-mg dose, maximal serum denosumab concentrations (C_{max}) were typically observed at 1 to 4 weeks postdose; after C_{max} serum denosumab levels declined over a period of 4 to 5 months with a mean half-life of approximately 25 to 30 days. The bioavailability of denosumab was approximately 60% after SC dosing. No accumulation in serum denosumab concentrations was observed with repeated doses of 60 mg Q6M. After repeated doses of 120 mg Q4W, an approximate 2-fold accumulation was observed, as expected based on denosumab's single-dose pharmacokinetic profile, and steady-state

was achieved by 6 months. Denosumab pharmacokinetics did not appear to change with time (up to 4 years exposure). The pharmacokinetic profile of denosumab was not notably affected by age, body mass index (BMI), sex, race (based on noncompartmental and population pharmacokinetic analysis methods), or renal function. The pharmacokinetic (PK) profile was associated with body weight; however, the correlation was independent of ethnicity and resulted in no clinically significant difference in pharmacodynamics.

For additional information on denosumab, please refer to the Clinical Investigator's Brochure [Denosumab (AMG 162) Investigator's Brochure, Edition 2.0, 23 August 2013].

1.2. Study Rationale

The purpose of this study is to provide safety, tolerability, pharmacokinetic and pharmacodynamic data for denosumab in healthy Chinese volunteers in order to meet regulatory requirements for the registration of denosumab.

1.3. Dose Rationale

The active doses proposed in this study are 60 mg and 120 mg. These doses have been shown to be safe and well tolerated. Our selection of these two doses is based on the following:

- Doses of 60 mg and 120 mg represent the doses proposed for approval in China: the 60 mg dose has been approved for the treatment of osteoporosis in postmenopausal women and the treatment of bone loss associated with hormone ablation in men with prostate cancer and in women with breast cancer in many other countries and regions; the 120 mg dose has been approved for prevention of skeletal related events (SRE) in patients with bone metastases from solid tumors;
- The doses of 60 mg and 120 mg have previously been assessed in healthy volunteers for safety, tolerability, pharmacokinetics and pharmacodynamics (s-CTX1 response).

1.4. Summary of Risk Management

Denosumab has been generally well tolerated in all clinical studies conducted to date.

Adverse events from previous clinical studies that have been considered by an investigator to be related to denosumab treatment have generally been diverse and mild to moderate in severity. The incidence of AEs, including serious adverse events (SAEs), was similar between subjects who received denosumab and those who received the active comparator or placebo treatment.

Identified and potential risks of denosumab treatment and management of these risks are included in the table below:

Risk	Impact on eligibility criteria	Monitoring
Osteonecrosis of the Jaw (ONJ)	Exclusion criteria:<br><br>- Prior history or current evidence of osteomyelitis or ONJ<br><br>- Active dental or jaw condition that requires oral surgery<br><br>- Planned invasive dental procedure for the course of the study<br><br>- Non-healed dental or oral surgery	Monitor oral AE events. All subjects with an oral AE suspicious of ONJ should be examined by a dentist or other qualified oral specialist. Oral AEs reported as or suspected to be ONJ will be reviewed by an independent adjudication panel of experts.
Atypical Femoral Fracture (AFF)	None	Monitor femoral fracture events. AFF reports will be reviewed by an independent adjudication panel of experts.
Transient hypocalcemia (seen in some Dmab-treated subjects)	Exclusion criterion:<br><br>Abnormal serum calcium at screen	Monitor serum calcium levels<br><br>Supplementation with calcium and vitamin D during the study
Immunogenicity (theoretical risk of MAbs)	None	Collection of antibody samples in ongoing studies
Hypersensitivity	Exclusion of subjects with a history of sensitivity to any of the study medications, especially known sensitivity to mammalian-derived drug preparations, or components thereof or a history of drug or other allergy that, in the opinion of the investigator or GSK Medical Monitor, contraindicates their participation	Monitoring post-dosing

Additional potential risks seen in previous studies of denosumab treatment include skin infections leading to hospitalization and eczema. Other theoretical risks include fracture healing complications. Should any of these events occur during the course of the study, the event will be evaluated and recorded as an AE/SAE (as applicable) and followed up by routine pharmacovigilance procedures (see Section 12).

Detailed information on safety is provided in the Investigator's Brochure [Denosumab (AMG 162) Investigator's Brochure, Edition 2.0, 23 August 2013].

2. OBJECTIVE(S)

2.1. Primary

- To assess the safety and tolerability of denosumab in healthy, adult Chinese subjects

2.2. Secondary

- To characterize the pharmacokinetic and pharmacodynamic properties of denosumab after single SC administration in healthy, adult Chinese subjects

3. ENDPOINT(S)

3.1. Primary

- Subject incidence of treatment-emergent adverse events, including clinically-significant changes in physical examinations, laboratory safety tests, ECG and vital signs

3.2. Secondary

- PK and PD (s-CTX1) parameter estimates

4. INVESTIGATIONAL PLAN

4.1. Study Design/Schematic

This is a randomized, parallel-group, single-blind, placebo-controlled study which will be conducted in a total of approximately 64 healthy volunteer subjects to assess the safety and tolerability of denosumab. Subjects will be randomized to denosumab 60 mg, denosumab 120 mg or placebo in a ratio of 3:3:2. Approximately 24 subjects will be randomized to each active treatment group and 16 to placebo in order to complete approximately 20 subjects for each active treatment group.

Reasonable diligence will be used to enrol about an equal number of males as females.

Figure 1 Study Design Schematic

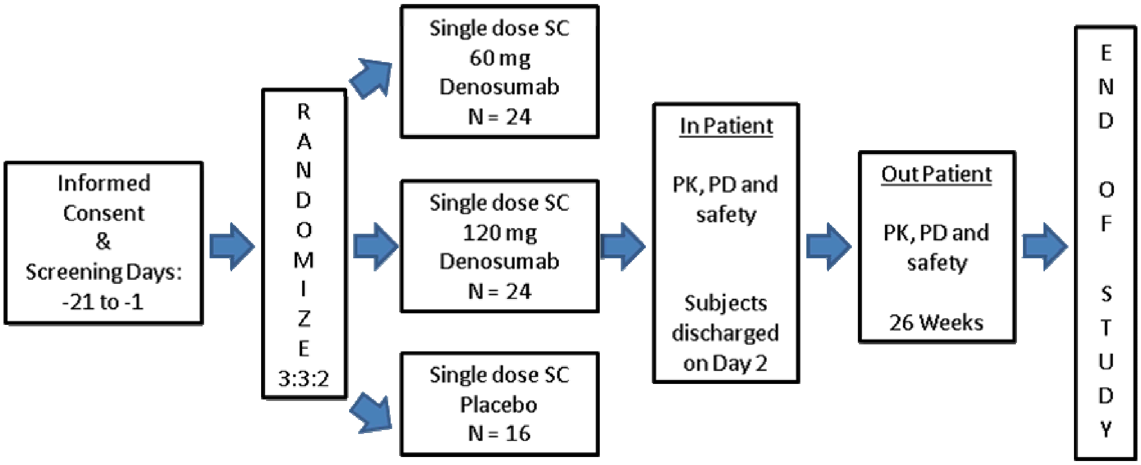

4.1.1. Screening

To determine subject eligibility for enrollment in the study, a Screening visit will be performed within 21 days of first dose of study drug. Screening assessments are defined as any assessments performed prior to the first dose of study drug including baseline assessments that are used to qualify the subject for enrollment.

4.1.2. Treatment

Subjects will be admitted to the clinic on Day -1 and will remain in-clinic until the morning of Day 2. On Day -1, baseline safety assessments including physical examinations (including oral examination), vital signs, ECG, and clinical chemistry, hematology and urinalysis will be made. The procedures will be repeated periodically throughout the treatment period. Refer to Section 4.5 Time and Events Table for details on the frequency of study assessments.

On Day 1, subjects will be randomized to receive either study medication or placebo. Subjects who receive study drug will receive either one injection of 60 mg denosumab + one injection of placebo or 2 injections of 60 mg (for a total of 120 mg) subcutaneously. Subjects randomized to placebo will receive matching placebo injections.

Subjects will remain in the clinic overnight on Day 1 for safety observation and will check out of the clinic on Day 2. Subjects will return to the clinic for study visits and safety assessments on days 3, 4, 5, 6, 8, 11 and at weeks 3, 4, 5, 7, 9, 11, 13, 15, 17, and 19. At week 26, a follow-up assessment will be conducted via telephone to identify any adverse events. Refer to Section 4.5 Time and Events Table for details.

All subjects will be provided with daily vitamin D (at least 400 IU but < 1000 IU/day) and calcium (at least 600 mg) supplementation for the duration of the study.

4.2. Discussion of Design

This study employs a randomized, single-blind, parallel, placebo-controlled design. The design employed for this study is identical to the design of the previous Phase 1 studies conducted by Amgen (Amgen Protocol Number 20060446, 2007).

This study includes a placebo arm to allow for a valid evaluation of adverse events attributable to treatment versus those independent of treatment.

Refer to Section 1.2 and Section 1.3 for additional information on the study and dose rationale.

4.3. Treatment Assignment

Subjects will be assigned to Denosumab 60 mg, Denosumab 120 mg or placebo in accordance with the randomization schedule generated by Discovery Biometrics, prior to the start of the study, using validated internal software.

A description of each regimen is provided in the table below:

Table 1 Dosing Regimen

Regimen	Description
A	Denosumab 60 mg + Denosumab 60 mg matched Placebo
B	Denosumab 60 mg + Denosumab 60 mg
P	Denosumab 60 mg matched Placebo + Denosumab 60 mg matched Placebo

4.4. Investigational Product Dosage/Administration

	Investigational Product	
**Product name:**	Denosumab	Placebo
**Dosage form:**	60 mg in 1.0mL solution	1.0 mL solution
**Unit dose strength(s)/Dosage level(s):**	Single use pre-filled syringes containing 1.0 mL solution of concentration 60 mg/mL	Single use pre-filled syringes containing 1.0 mL solution
**Route/ Administration/ Duration:**	Subcutaneous injections in subjects' anterior abdominal wall	Subcutaneous injections in subjects' anterior abdominal wall
**Dosing instructions:**	Each subject will receive 2 subcutaneous injections on Day 1 per the randomization schedule	Each subject will receive 2 subcutaneous injections on Day 1 per the randomization schedule
**Manufacturer/ source of procurement:**	Amgen Inc.	Amgen Inc.

4.4.1. Dose Adjustment/Stopping Safety Criteria

This is a single dose study and subjects will be in-house for safety monitoring for at least 24 hours following the dose of study medication. Subjects will also return for safety assessments daily and then weekly for 19 weeks. Dose adjustment will not be possible

for individual subjects. However, potential safety signals will be closely monitored by the GSK Medical Monitor across subjects throughout the duration of the study and any findings that arise will be fully interrogated.

4.5. Time and Events Table

Day:	Screening	d-1	d1	d2	d3	d4	d5	d6	d8	d11	d21	d28	d35	d49	d63	d77	d91	d105	d119	d133 / early withdrawal	d182 Follow-up (via Phone)
**Week:**									w2		w3	w4	w5	w7	w9	w11	w13	w15	w17	w19	w26
**Visit Window** (relative to Day 1)	-21 to -1 days	NA									±2 days										
**Admission to Unit**		X																			
**Informed Consent**	X																				
**Inclusion/Exclusion**	X	X																			
**Demographics**	X																				
**Physical Examination**	X	X																		X	
**Medical history**	X																				
**12-lead ECG**	X[1]	X																		X	
**Chest X Ray Exam**		X																			
**Vital signs(BP and pulse rate)**	X	X	X[2]	X	X	X	X	X	X	X	X	X	X	X	X	X	X	X	X	X	
**Height**	X																				
**Weight**	X																			X	
**Urine drug/alc. screen**	X	X																			
**Urine pregnancy test (women)**		X											X				X			X	
**Serum β-hCG pregnancy test (women)**	X																				
**HIV, Hep B and Hep C, Syphilis screen**	X																				
**Hema/Chem/Urinalysis**	X	X[6]							X[3]				X				X			X	
**AE assessment**	X[4]	X[4]	X	X	X	X	X	X	X	X	X	X	X	X	X	X	X	X	X	X	X
**Pregnancy assessment phone call**																					X
**Conmed Review**			X	X	X	X	X	X	X	X	X	X	X	X	X	X	X	X	X	X	
**PK blood sample**			X[5]	X[5]	X[5]	X	X	X	X	X	X	X	X	X	X	X	X	X	X	X	

Day:	Screening	d-1	d1	d2	d3	d4	d5	d6	d8	d11	d21	d28	d35	d49	d63	d77	d91	d105	d119	d133 / early withdrawal	d182 Follow-up (via Phone)
**Week:**									w2		w3	w4	w5	w7	w9	w11	w13	w15	w17	w19	w26
**Visit Window** (relative to Day 1)	-21 to -1 days	NA									±2 days										
**PD (s-CTX1) sample**			X[2]	X	X	X	X	X	X	X	X	X	X	X	X	X	X	X	X	X	
**Antibody sample**			X[2]									X			X					X	
**Dosing**			X																		
**Discharge from Unit**				X																	
**Outpatient visit**	X				X	X	X	X	X	X	X	X	X	X	X	X	X	X	X	X	

1. Triplicate ECG at Screening to confirm eligibility. Single ECG at other time points.
2. Assessments performed prior to dosing.
3. Only serum calcium is required on Day 8.
4. Only SAEs related to study participation will be recorded from the signing of consent to study drug administration.
5. PK blood samples will be drawn pre-dose, 1hr, 4 hrs, 8 hrs, 12 hrs, 24 hrs (Day 2), and 48 hrs (Day 3) post-dose.
6. If Screening visit is within 14 days prior to Day -1, then Hema/Chem/Urialysis tests at Day -1 can be exemption.

5. STUDY POPULATION

5.1. Number of Subjects

Approximately 64 subjects will be enrolled such that approximately 20 subjects per active dose group and 14 subjects in the placebo group complete dosing and critical assessments.

5.2. Eligibility Criteria

5.2.1. Inclusion Criteria

A subject will be eligible for inclusion in this study only if all of the following criteria apply:

1. Resident in China and of Chinese ancestry.
2. AST, ALT, alkaline phosphatase and bilirubin ≤ 1.5xULN (isolated bilirubin > 1.5xULN is acceptable if bilirubin is fractionated and direct bilirubin < 35%).
3. Healthy as determined by a responsible and experienced physician, based on a medical evaluation including medical history, physical examination, laboratory tests and cardiac monitoring. A subject with a clinical abnormality or laboratory parameters outside the reference range for the population being studied may be included only if the Investigator and the GSK Medical Monitor agree that the finding is unlikely to introduce additional risk factors and will not interfere with the study procedures.
4. Male or female between 18 and 65 years of age, inclusive, from date of birth, at the time of signing the informed consent.
5. A female subject is eligible to participate if she is of:
   - Non-childbearing potential defined as pre-menopausal females with a documented tubal ligation or hysterectomy; or postmenopausal defined as 12 months of spontaneous amenorrhea. In questionable cases a blood sample with simultaneous follicle stimulating hormone (FSH) > 40 MlU/ml and estradiol < 40 pg/ml (< 140 pmol/L) is confirmatory.

     OR
   - Child-bearing potential and agrees to use one of the contraception methods listed in Section 8.1 for an appropriate period of time (as determined by the product label or investigator) prior to the start of dosing to sufficiently minimize the risk of pregnancy at that point. Female subjects must agree to use contraception for the duration of the study and for a minimum of 6 months after the last dose of study medication.
6. Body weight of at least 50 kg and body mass index (BMI) from 19 to 24 kg/m^2 at time of screening.
7. Capable of giving written informed consent, which includes compliance with the requirements and restrictions listed in the consent form.
8. Average QTcB or QTcF < 450 msec; or QTc < 480 msec in subjects with Bundle Branch Block.

5.2.2. Exclusion Criteria

A subject will not be eligible for inclusion in this study if any of the following criteria apply:

1. A prior history or current evidence of osteomyelitis or ONJ.
2. An active dental or jaw condition that requires oral surgery.
3. A planned invasive dental procedure during the course of the study.
4. A non-healed dental or oral surgery.
5. A positive pre-study Hepatitis B surface antigen or positive Hepatitis C antibody result within 3 months of screening.
6. Current or chronic history of liver disease, or known hepatic or biliary abnormalities (with the exception of Gilbert's syndrome or asymptomatic gallstones).
7. A positive pre-study drug/alcohol screen.
8. A positive test for HIV antibody.
9. A positive test for syphilis at Screening.
10. Abnormal serum calcium: current hypocalcemia or hypercalcemia. Albumin-adjusted serum calcium levels must be within the normal range of the central laboratory.
11. History of regular alcohol consumption within 6 months of the study defined as an average weekly intake of > 14 drinks/week for men or > 7 drinks/week for women. One drink is equivalent to (12 g alcohol) = 150 ml (5 ounces) of table wine or 360 ml (12 ounces) of beer or 45 ml (1.5 ounces) of 80 proof distilled spirits.
12. The subject has participated in a clinical trial and has received an investigational product within the following time period prior to the first dosing day in the current study: 30 days, 5 half-lives or twice the duration of the biological effect of the investigational product (whichever is longer).
13. Exposure to more than four new chemical entities within 12 months prior to the first dosing day.
14. History of sensitivity to any of the study medications, especially known sensitivity to mammalian-derived drug preparations, or components thereof or a history of drug or other allergy that, in the opinion of the investigator or GSK Medical Monitor, contraindicates their participation.
15. Where participation in the study would result in donation of blood or blood products in excess of 500 mL within a 56 day period.
16. Pregnant females as determined by positive serum human chorionic gonadotropin (hCG) test at screening or urine hCG test prior to dosing (day -1).
17. A chest X-ray or computed tomography (CT) scan that reveals evidence of clinical significant abnormalities e.g., tuberculosis. A chest X-ray must be taken at Day-1 if a chest X-ray or CT scan is not available within 6 months prior to that day.
18. Lactating females.

19. Unwillingness or inability to follow the procedures outlined in the protocol.
20. Subject is mentally or legally incapacitated.
21. History of sensitivity to heparin or heparin-induced thrombocytopenia.
22. Significant changes in physical activity during the 6 months before study drug administration or constant levels of intense physical exercise.
23. Prior use of medications within 4 weeks or 5 half-lives (whichever period is greater) before and during the study. This includes medications such as, but not limited to:
    - Estrogen-containing contraceptives
    - Bisphosphonates
    - Fluoride
    - Hormone replacement therapy (i.e., tibolone, estrogen, estrogen-like compounds such as raloxifene)
    - Calcitonin
    - Strontium
    - Parathyroid hormone or derivatives
    - Supplemental vitamin D (>1000 IU/day)
    - Glucocorticosteroids (inhaled or topical corticosteroids administered more than 2 weeks prior to enrolment are allowed)
    - Anabolic steroids
    - Calcitriol
    - Diuretics

5.2.3. Other Eligibility Criteria Considerations

To assess any potential impact on subject eligibility with regard to safety, the investigator must refer to the following document(s) for detailed information regarding warnings, precautions, contraindications, adverse events, and other significant data pertaining to the investigational product(s) being used in this study: [Denosumab (AMG 162) Investigator's Brochure, Edition 2.0, 23 August 2013].

6. DATA ANALYSIS AND STATISTICAL CONSIDERATIONS

6.1. Hypotheses and Treatment Comparisons

This study is designed to characterize the pharmacokinetic and pharmacodynamic properties of denosumab after single SC administration. No formal hypothesis will be tested. For each pharmacokinetic endpoint, point estimates and corresponding 90% confidence intervals will be constructed.

6.2. Sample Size Considerations

6.2.1. Sample Size Assumptions

Approximately 64 subjects will be enrolled so that approximately 54 subjects (20 subjects for 60 mg group, 20 subjects for 120 mg group, and 14 subjects for placebo group) complete the study. Sample size is based in part on feasibility. However, some justification is provided below.

Based on results from Amgen study 20050146(Amgen Protocol Number 20050146, 2006), the between subject CV for denosumab AUC and Cmax are 37% and 32% respectively. Based on 37% CV, it is estimated that the half width of 90% confidence interval will be approximately 14.9% of the point estimate with 20 subjects. Based on Amgen Protocol Number 20050146, 2006, the between subject CV for s-CTX1 AUEC and Imax are 14% and 10% respectively. Based on 14% CV, it is estimated that the half width of 90% confidence interval will be approximately 5.6% of the point estimate with 20 subjects.

6.2.2. Sample Size Sensitivity

Assuming similar between subject CV as above, it is estimated that the PK half width of 90% confidence interval will be approximately 17.0% of the point estimate with 16 subjects. And it is estimated that the PD half width of 90% confidence interval will be approximately 6.3% of the point estimate with 16 subjects.

6.2.3. Sample Size Re-estimation

No sample size re-estimation will be performed.

6.3. Data Analysis Considerations

6.3.1. Interim Analysis

No interim analysis is planned.

6.3.2. Final Analyses

6.3.2.1. Safety Analyses

Safety data will be presented in tabular and/or graphical format and summarized descriptively according to GSK's Integrated Data Standards Library (IDSL) standards.

6.3.2.2. Pharmacokinetic Analyses

Pharmacokinetic analysis will be the responsibility of the Clinical Pharmacology Modeling & Simulation (CPMS) department within GlaxoSmithKline. Plasma GSK2371746 concentration-time data will be analyzed by non-compartmental methods with WinNonlin 5.2 or above. Calculations will be based on the actual sampling times recorded during the study. From the plasma concentration-time data, the following pharmacokinetic parameters will be determined, as data permit: maximum observed

plasma concentration (Cmax), time to Cmax (tmax), and area under the plasma concentration-time curve AUC(0-t). Pharmacokinetic data will be presented in graphical and/or tabular form and will be summarized descriptively. All pharmacokinetic data will be stored in the Archives, GlaxoSmithKline Pharmaceuticals, R&D.

Plasma GSK2371746 concentration-time data will be analyzed by nonlinear mixed modeling methods with NONMEM 6.2 or above. Calculations will be based on the actual sampling times recorded during the study. Plasma pharmacokinetic profiles will be explained and predicted for actual subject demographics, doses and sampling times from a pharmacometric model based on the Complete Clinical Data Package, and the predicted profiles will be compared with the observed profiles. The comparison between predicted and observed profiles will be presented in graphical and/or tabular form and will be summarized descriptively. Details of the model will be provided in the Reporting Analysis Plan.

Statistical analyses of the pharmacokinetic parameter data will be the responsibility of Discovery Biometrics, GlaxoSmithKline.

Pharmacokinetic parameters will be summarized by treatment group. Mean, Median, Min, Max, SD, GeoMean, log SD, CV, 90% CI will be provided; Individual and mean PK concentration-time curves will be provided.

6.3.2.3. Pharmacokinetic/Pharmacodynamic Analyses

Serum CTX1 concentration-time data will be analyzed by nonlinear mixed modeling methods with NONMEM 6.2 or above. Calculations will be based on the actual sampling times recorded during the study. Serum CTX1 pharmacodynamic profiles will be explained and predicted for actual subject demographics, doses and sampling times from a pharmacometric model based on the Complete Clinical Data Package, and the predicted profiles will be compared with the observed profiles. The comparison between predicted and observed profiles will be presented in graphical and/or tabular form and will be summarized descriptively. Details of the model will be provided in the Reporting Analysis Plan.

6.3.2.4. Pharmacodynamic/Biomarker Analyses

Pharmacodynamic/Biomarker analysis will be the responsibility of the Clinical Pharmacokinetics Modeling & Simulation department within GlaxoSmithKline. Serum CTX1 effect-time data will be analyzed by non-compartmental methods with WinNonlin 5.2. Calculations will be based on the actual sampling times recorded during the study. From the plasma effect-time data, the following pharmacodynamic parameters will be determined, as data permit: minimum observed plasma concentration (Imin), time to Imin (tmin_s-CTX1), and area under the plasma effect-time curve AUEC(0-t). Pharmacodynamic data will be presented in graphical and/or tabular form and will be summarized descriptively. All pharmacodynamic data will be stored in the Archives, GlaxoSmithKline Pharmaceuticals, R&D.

Statistical analyses of the pharmacodynamic parameter data will be the responsibility of Discovery Biometrics, GlaxoSmithKline.

PD parameters (serum CTX1 AUEC and Imax) will be summarized by treatment group; Mean, Median, Min, Max, SD, GeoMean, log SD, CV, 90% CI will be provided; Individual and mean s-CTX1 inhibition curves will be provided.

6.3.2.5. Efficacy Analyses

No efficacy analysis is planned.

7. STUDY ASSESSMENTS AND PROCEDURES

This section lists the parameters of each planned study assessment. The exact timing of each assessment is listed in the Time and Events Table (Section 4.5). Detailed procedures for obtaining each assessment are provided in the Study Procedures Manual (SPM). Whenever vital signs, 12-lead ECGs and blood draws are scheduled for the same nominal time, the assessments should occur in the following order: 12-lead ECG, vital signs, and then blood draws. The timing of the assessments should allow the blood draw to occur at the exact nominal time.

The timing and number of planned study assessments, including safety, pharmacokinetic, pharmacodynamic/biomarker or others assessments may be altered during the course of the study based on newly available data to ensure appropriate monitoring. The change in timing or addition of time points for any planned study assessments must be approved and documented by GSK, but this will not constitute a protocol amendment. The IRB/IEC will be informed of any safety issues that require alteration of the safety monitoring scheme. There will be 22 time points for PK blood sample collection and approximately 3.5 mL of blood will be collected each time. There will be 18 time points for PD blood sample collection and approximately 3.5 mL of blood will be collected each time. Haematological and chemical assessment will be tested for 5 times, approximately 6.5 mL of blood will be collected for each test. Virological assessment will be tested at screening and approximately 3 mL of blood will be collected. Blood pregnancy test will be tested in female subjects at screening, approximately 1.5 mL of blood will be collected . Denosumab antibody will be tested for 4 times and 7 mL of blood will be collected each time. Approximately 200 mL of blood will be collected over the duration of the study. If Investigator consider that additional safety assessment is needed, additional blood samples may be required.

7.1. Demographic/Medical History Assessments

Each subject will have adequate time to provide informed consent of his or her own free will, and prior to conducting any study related procedures, according to the International Conference on Harmonization (ICH) and the US Code of Federal Regulations (CRF) guidelines. Each subject will be provided with a copy of the signed and dated informed consent form prior to the initiation of any study procedures.

After a subject has provided written informed consent, and within 21 days of dosing, the Principal Investigator or other study personnel will determine if the subject is eligible for enrollment in the study. This will be done by reviewing the inclusion and exclusion criteria and completing all of the Screening assessments outlined in Section 4.5. Screening assessments may be carried out over more than one day provided that all

required assessments are completed prior to Day -1. Screening labs may be repeated at the investigator's discretion if, for example, lab results indicate that the subject was not appropriately fasted. Labs obtained during the treatment period may be repeated at the investigator's discretion.

The following demographic parameters will be captured: year of birth, gender, race and ethnicity.

Medical/medication/alcohol history will be assessed as related to the eligibility criteria listed in Section 5.2.

7.2. Safety

The investigator or other qualified study personnel should conduct the safety assessments described in this section and in Section 12 (Adverse Events and Serious Adverse Events).

Subjects should fast from midnight prior to collection of blood samples for clinical laboratory testing.

Planned timepoints for all safety assessments are listed in the Time and Events Table (Section 4.5). Additional time points for safety tests (such as vital signs, physical exams and laboratory safety tests) may be added during the course of the study based on newly available data and/or to ensure appropriate safety monitoring.

7.2.1. Physical Examinations

A complete physical examination will include assessments of the head, eyes, ears, nose, mouth, throat, skin, thyroid, neurological, lungs, cardiovascular, abdomen (liver and spleen), lymph nodes and extremities. Height and weight will also be measured and recorded.

7.2.2. Vital Signs

Vital sign measurements will include systolic and diastolic blood pressure and pulse rate.

At each timepoint, assessments should be performed after the subject has been resting in a supine/semi-supine position for at least 5 minutes.

7.2.3. Height

Standing height should be measured in centimeters. Subjects must remove their shoes prior to obtaining the measurement.

7.2.4. Weight

Body weight will be obtained in kilograms (kg). Subjects should be weighed on the same scale at every visit after the screening visit. Lightweight indoor clothing should be worn and shoes must be removed.

7.2.5. Body Mass Index

BMI will be determined by the formula BMI = [weight in kg/(height in meters)^2].

7.2.6. Electrocardiogram (ECG)

12-lead ECGs will be obtained at each timepoint (refer to Section 4.5 Time and Events Table) during the study using an ECG machine that automatically calculates the heart rate and measures PR, QRS, QT, and QTc intervals.

ECGs should be performed after the subject has rested in a supine/semi-supine position for at least 5 minutes. Additional ECGs may be taken at the discretion of the investigator as needed based on signs or symptoms.

7.2.7. Clinical Laboratory Assessments

Hematology, clinical chemistry, urinalysis and additional parameters to be tested are listed below:

Hematology

Platelet Count	*RBC Indices*:	*Automated WBC Differential:*
RBC Count	MCV	Neutrophils
WBC Count (absolute)	MCH	Lymphocytes
Hemoglobin	MCHC	Monocytes
Hematocrit		Eosinophils
		Basophils

Clinical Chemistry

BUN	Potassium	AST	Total bilirubin
Creatinine	Chloride	ALT	Direct bilirubin
Glucose, fasting	GGT	Total Protein	Albumin
Sodium	Calcium	Alkaline phosphatase	

NOTE: Details of Liver Chemistry Follow-up Procedures are given in Section 13.

Routine Urinalysis

Specific gravity
pH, glucose, protein, blood, bilirubin and ketones by dipstick
Microscopic examination (if blood or protein is abnormal)

Other screening tests

HIV
Syphilis (syphilis antibody)
Serum hCG test at screening, urine hCG test prior to dosing (day -1)
Hepatitis B (HBsAg)
Hepatitis C (Hep C antibody)
FSH and estradiol (as needed in women of non-child bearing potential only)
Alcohol and drug screen (to include at minimum: amphetamines, barbiturates, cocaine, opiates, cannabinoids and benzodiazepines).

7.3. Pregnancy

7.3.1. Time period for collecting pregnancy information

Information on all pregnancies in female subjects and/or female partners of male subjects will be collected after the start of dosing and until 6 months post last dose. These events will be reported to Amgen's existing Pregnancy Surveillance Program.

7.3.2. Action to be taken if pregnancy occurs

Any pregnancy that occurs during study participation must be reported using a clinical trial pregnancy form. To ensure subject safety, each pregnancy must be reported to GSK within two weeks of learning of its occurrence. The pregnancy must be followed up to determine outcome (including premature termination) and status of mother and child. Pregnancy complications and elective termination for medical reasons must be reported as an AE or SAE. Spontaneous abortions must be reported as an SAE.

Any SAE occurring in association with a pregnancy brought to the Investigator's attention after the subject has completed the study and considered by the Investigator as possibly related to the investigational product, must be promptly reported to GSK.

A spontaneous abortion is always considered to be an SAE and will be reported as such. Furthermore, any SAE occurring as a result of a post-study pregnancy and is considered reasonably related to the investigational product by the investigator, will be reported to GSK as described in Section 12. While the investigator is not obligated to actively seek this information in former study participants, he or she may learn of an SAE through spontaneous reporting.

Any female subject who becomes pregnant while participating will continue participating in the study.

7.3.3. Action to be taken if pregnancy occurs in a female partner of a male study subject

The investigator will attempt to collect pregnancy information on any female partner of a male study subject who becomes pregnant while participating in this study. The investigator will record pregnancy information on the appropriate form and submit it to GSK within 2 weeks of learning of the partner's pregnancy. The partner will also be followed to determine the outcome of the pregnancy. Information on the status of the mother and child will be forwarded to GSK. Generally, follow-up will be no longer than 6 to 8 weeks following the estimated delivery date. Any premature termination of the pregnancy will be reported.

7.4. Pharmacokinetics

7.4.1. Blood Sample Collection

Blood samples (approximately 3.5 mL) for pharmacokinetic analysis of GSK2371746 will be collected at the time points indicated in Section 4.5 Time and Events Table. The

actual date and time of each blood sample collection will be recorded. The timing of PK samples may be altered and/or PK samples may be obtained at additional time points to ensure thorough PK monitoring.

Details of PK blood sample processing, storage and shipping procedures are provided in the Study Procedures Manual (SPM).

7.4.2. Sample Analysis

Concentration determination of GSK2371746 in serum will be performed by Wuxi AppTec using a validated bioanalytical methodology. Raw data will be stored in the Good Laboratory Practices Archives at Wuxi AppTec for five years before sponsor transfer request. Further details on methodology and sample analysis results will be included in the method validation and sample analysis reports.

7.5. Biomarker(s)/Pharmacodynamic Markers

7.5.1. Confirmed Biomarkers/Pharmacodynamic Markers

s-CTX1 is selected as pharmacodynamic marker in this study. Blood samples (approximately 3.5 mL) should be taken in the morning after an overnight fast. Serum samples will be assayed for s-CTX1 by the electrochemiluminescence immunosassay. Refer to the Study Procedures Manual for detailed instructions on collection and processing of these samples. No other biomarkers will be collected or analyzed during this study.

7.6. Denosumab Antibody (Seroreactivity) Assay

Blood samples (approximately 7 mL) for Denosumab antibodies (binding and neutralizing) analysis will be collected at the time points indicated in Section 4.5, Time and Events Table. Denosumab antibody analysis will be assayed by Frontage. If the serum sample is positive for an antibody to denosumab, serum sample(s) will be exported to the laboratory in Amgen for examination in a confirmatory assay. Refer to the Study Procedures Manual for detailed instructions on collection and processing of these samples.

8. LIFESTYLE AND/OR DIETARY RESTRICTIONS

8.1. Contraception Requirements

8.1.1. Female Subjects

Female subjects of childbearing potential must not become pregnant and so must be sexually inactive by abstinence or use contraceptive methods with a failure rate of < 1% throughout the study and for a minimum of 6 months after last dose of investigational product.

Abstinence

Sexual inactivity by abstinence must be consistent with the preferred and usual lifestyle of the subject. Periodic abstinence (e.g., calendar, ovulation, symptothermal, post-ovulation methods) and withdrawal are not acceptable methods of contraception.

Contraceptive Methods with a Failure Rate of < 1%

- Oral contraceptives with progestogen alone
- Injectable progestogen
- Implants of levonogestrel
- Intrauterine device (IUD) or intrauterine system (IUS) that meets the < 1% failure rate as stated in the product label
- Male partner sterilization (vasectomy with documentation of azoospermia) prior to the female subject's entry into the study, and this male is the sole partner for that subject. For this definition, “documented” refers to the outcome of the investigator's/designee’s medical examination of the subject or review of the subject's medical history for study eligibility, as obtained via a verbal interview with the subject or from the subject’s medical records.
- Double barrier method: condom and occlusive cap (diaphragm or cervical/vault caps) plus spermicidal agent (foam/gel/film/cream/suppository)

These allowed methods of contraception are only effective when used consistently, correctly and in accordance with the product label. The investigator is responsible for ensuring subjects understand how to properly use these methods of contraception.

NOTE: All natural and synthetic estrogens are prohibited

8.2. Meals and Dietary Restrictions

Subjects should fast from all food or drink with the exception of water from midnight prior to each blood collection for clinical laboratory tests. Subjects should fast from all food or drink with the exception of water from midnight prior to administration of study drug. Water is permitted ad lib.

8.3. Caffeine, Alcohol, and Tobacco

Caffeine consumption during the study should not change substantially from pre-study levels for 24 hours prior to the start of dosing until collection of the final pharmacokinetic and or pharmacodynamic sample during the in-house portion of the study.

Alcohol consumption should not exceed an average weekly intake of 14 drinks/week for men or 7 drinks/week for women. One drink is equivalent to (12 g alcohol) = 150 ml (5 ounces) of table wine or 360 ml (12 ounces) of beer or 45 ml (1.5 ounces) of 80 proof distilled spirits.Subjects who use tobacco products will be instructed that use of nicotine-containing products (including nicotine patches) will not be permitted while they are in the Clinical Unit.

8.4. Activity

Subjects will abstain from strenuous exercise for 48 hours prior to each blood collection for clinical laboratory tests. Subjects may participate in light recreational activities while in the clinic (e.g., watch television, read).

9. CONCOMITANT MEDICATIONS AND NON-DRUG THERAPIES

9.1. Permitted Medications

Acetaminophen (Paracetamol) or Ibuprofen at doses of ≤ 2 grams/day is permitted. Other concomitant medication may be considered on a case by case basis by the GSK Medical Monitor.

Corticosteroids (inhaled or topical) administered more than 2 weeks prior to enrolment and continuing throughout the study will be permitted.

9.2. Prohibited Medications

Subjects must abstain from taking prescription or non-prescription drugs (including vitamins and dietary or herbal supplements), within 7 days (or 14 days if the drug is a potential enzyme inducer) or 5 terminal half-lives (whichever is longer) prior to the first dose of study medication until completion of the follow-up visit, unless in the opinion of the Investigator and sponsor the medication will not interfere with the study. These restrictions apply to prescribed medications such as, but not limited to, bisphosphonates, fluoride, hormone replacement therapies (i.e., estrogen, tibolone, and estrogen-like compounds such as raloxifene), calcitonin, strontium, parathyroid hormone, supplemental vitamin D (> 1000 IU/day), glucocorticoids, anabolic steroids, calcitriol, and diuretics.

Use of estrogen-containing contraceptives will not be permitted throughout this study. Subjects of childbearing potential must agree to the Contraceptive Requirements (Section 8.1).

Throughout study participation, subjects should limit alcohol intake to 2 drinks or less per day (one drink being equivalent to 360 ml (12 ounces) of regular beer, 150 ml (5 ounces) of wine, or 45 ml (1.5 ounces) of 80 proof distilled spirits).

Acetaminophen should not be used in patients with acute viral hepatitis.

9.3. Non-Drug Therapies

Subjects must abstain from taking any vitamins, herbal or dietary supplements, traditional Chinese medicines, and herbal teas within 7 days (or 14 days if the drug is a potential enzyme inducer) or 5 terminal half-lives (whichever is longer) prior to the first dose of study medication until completion of the follow-up visit, unless in the opinion of the Investigator and sponsor the medication will not interfere with the study.

10. COMPLETION OR EARLY WITHDRAWAL OF SUBJECTS

10.1. Subject Completion

A completed subject is one who has completed all phases of the study including the follow-up visit.

The end of the study is defined as the last subject's last visit.

10.2. Subject Withdrawal Criteria

A subject may withdraw from investigational product at any time at his/her own request, or may be withdrawn at any time at the discretion of the investigator for safety, behavioral or administrative reasons.

10.3. Subject Withdrawal Procedures

10.3.1. Subject Withdrawal from Study

Every effort should be made for the retention of subjects into the study. At the discretion of the investigator, a subject may be withdrawn from the study due to an AE, SAE, or fracture.

A subject may voluntarily discontinue participation in this study or may be withdrawn at the discretion of the investigator for safety or administrative reasons. A subject may also be withdrawn from the study for the following reasons:

- A significant protocol violation as determined by either the sponsor or investigator
- At the specific request of the sponsor
- Concomitant use of any investigational medication, other than study medication

If a subject is withdrawn from the study, perform all of the procedures described in the Time and Events Table for the early withdrawal visit (unless they have withdrawn informed consent).

10.4. Treatment After the End of the Study

Subjects will not receive any additional treatment after completion of the study because only healthy volunteers are eligible for study participation.

10.5. Screen and Baseline Failures

Data for screen and baseline failures will be collected in source documentation at the site. Minimal screen failure information will be collected on CRF, including Demography, Screen Failure details, Eligibility Criteria and Serious Adverse Events.

11. INVESTIGATIONAL PRODUCT(S)

11.1. Description of Investigational Product

The investigational product in this study, Denosumab (AMG 162; GSK2371746) 60 mg, SC, will be supplied as single use pre-filled syringes containing 1.0 mL solution of concentration 60 mg/mL denosumab or Placebo to match.

Each pre-filled syringe will be packed in a carton. The contents of the label will be in accordance with all applicable regulatory requirements. The supplies should be protected from light, stored in a safe and secure place in a refrigerator at 2-8 degrees. C (35.6-46.4 degrees F) and must not be frozen.

Investigators will provide calcium and vitamin D supplements to all subjects randomized into the study. The recommended administration of these supplements is at least 600 mg calcium (daily) and at least 400 IU vitamin D but <1000 IU daily for the duration of the study.

11.2. Handling and Storage

Drug supplies must be kept in an appropriate restricted area, which may be accessed only by the pharmacist, or a duly designated person.

If a breakdown of the refrigerator occurs, the supplies should be transferred to another temperature controlled refrigerator immediately and the local monitor should be contacted to assess the situation and process all appropriate documentation that may be required.

The supplies must not be utilized after the expiry date printed on the product label.

A log to document the temperature with daily readings must be maintained as a GCP requirement.

Investigational product dosage and administration details are listed in Section 4.4.

11.3. Blinding

This will be a single-blind study. The subjects will be blinded, GSK and site staff will be unblinded.

11.4. Packaging and Labeling

The contents of the label will be in accordance with all applicable regulatory requirements.

11.5. Preparation/Handling/Storage/Accountability

No special preparation of investigational product is required.

Investigational product must be dispensed or administered according to procedures described herein. Only subjects enrolled in the study may receive investigational product. Only authorized site staff may supply or administer investigational product. All investigational products must be stored in a secure area with access limited to the investigator and authorized site staff. Investigational product is to be stored at 2 to 8 degrees C (35.6 to 46.4 degrees F) and must not be frozen. Maintenance of a temperature log (manual or automated) is required.

The investigator, institution, or the head of the medical institution (where applicable) is responsible for investigational product accountability, reconciliation, and record maintenance. The investigator or the head of the medical institution (where applicable), or designated site staff (e.g., storage manager, where applicable) must maintain investigational product accountability records throughout the course of the study. The responsible person(s) will document the amount of investigational product received from and returned to GSK and the amount administered to subjects. The required accountability unit for this study will be syringe. Discrepancies are to be reconciled or resolved. Procedures for final disposition of unused investigational product are listed in the SPM.

Investigational product is not expected to pose significant occupational safety risk to site staff under normal conditions of use and administration. A Material Safety Data Sheet (MSDS)/equivalent document describing occupational hazards and recommended handling precautions either will be provided to the investigator, where this is required by local laws, or is available upon request from GSK.

However, precautions are to be taken to avoid direct skin contact, eye contact, and generating aerosols or mists. In the case of unintentional occupational exposure notify the monitor, GSK Medical Monitor and/or study manager.

11.6. Assessment of Compliance

When subjects are dosed at the study site, they will receive investigational products directly from the investigator or designee, under medical supervision. The date and time of each dose administered in the clinic will be recorded in the source documents. The dose of investigational product(s) and study participant identification will be confirmed at the time of dosing by a member of the study site staff other than the person administering the investigational product.

11.7. Treatment of Investigational Product Overdose

Overdose of study medication is unlikely to occur in this study, as dosing of **all** study medications will be supervised at the treatment visit.

GSK does not recommend specific treatment for an overdose. The investigator will use clinical judgment to treat any overdose.

12. ADVERSE EVENTS (AE) AND SERIOUS ADVERSE EVENTS (SAE)

The investigator or site staffare responsible for detecting, documenting and reporting events that meet the definition of an AE or SAE.

AEs will be collected from the start of Investigational Product and until the follow-up contact. Medical occurrences that begin prior to the start of investigational product but after obtaining informed consent may be recorded on the Medical History/Current Medical Conditions CRF.

SAEs will be collected over the same time period as stated above for AEs. However, any SAEs assessed as related to study participation (e.g., investigational product, protocol-mandated procedures, invasive tests, or change in existing therapy) or related to a GSK concomitant medication will be recorded from the time a subject consents to participate in the study up to and including any follow-up contact. All SAEs will be recorded and reported to GSK within 24 hours, as indicated in Section 12.9.

Investigators are not obligated to actively seek AEs or SAEs in former study participants. However, if the investigator learns of any SAE, including a death, at any time after a subject has been discharged from the study, and he/she considers the event reasonably related to the investigational product or study participation, the investigator would promptly notify GSK.

12.1. Definition of Adverse Events

An AE is any untoward medical occurrence in a patient or clinical investigation subject, temporally associated with the use of a medicinal product, whether or not considered related to the medicinal product.

Note: An AE can therefore be any unfavorable and unintended sign (including an abnormal laboratory finding), symptom, or disease (new or exacerbated) temporally associated with the use of a medicinal product.

Events meeting the definition of an AE **include**:

- Any abnormal laboratory test results (hematology, clinical chemistry, or urinalysis) or other safety assessments (e.g., ECGs, radiological scans, vital signs measurements), including those that worsen from baseline, and felt to be clinically significant in the medical and scientific judgement of the investigator.
- Exacerbation of a chronic or intermittent pre-existing condition including either an increase in frequency and/or intensity of the condition.
- New conditions detected or diagnosed after investigational product administration even though it may have been present prior to the start of the study.
- Signs, symptoms, or the clinical sequelae of a suspected interaction.

- Signs, symptoms, or the clinical sequelae of a suspected overdose of either investigational product or a concomitant medication (overdose *per se* will not be reported as an AE/SAE).

Events that **do not** meet the definition of an AE include:

- Medical or surgical procedure (e.g., endoscopy, appendectomy); the condition that leads to the procedure is an AE.
- Situations where an untoward medical occurrence did not occur (social and/or convenience admission to a hospital).
- Anticipated day-to-day fluctuations of pre-existing disease(s) or condition(s) present or detected at the start of the study that do not worsen.

12.2. Definition of Serious Adverse Events

If an event is not an AE per Section 12.1, then it cannot be an SAE even if serious conditions are met (e.g., hospitalization for signs/symptoms of the disease under study, death due to progression of disease, etc).

An SAE is any untoward medical occurrence that, at any dose:

a. Results in death

b. Is life-threatening

   Note: The term 'life-threatening' in the definition of 'serious' refers to an event in which the subject was at risk of death at the time of the event. It does not refer to an event, which hypothetically might have caused death, if it were more severe.

c. Requires hospitalization or prolongation of existing hospitalization

   Note: In general, hospitalization signifies that the subject has been detained (usually involving at least an overnight stay) at the hospital or emergency ward for observation and/or treatment that would not have been appropriate in the physician's office or out-patient setting. Complications that occur during hospitalization are AEs. If a complication prolongs hospitalization or fulfills any other serious criteria, the event is serious. When in doubt as to whether "hospitalization" occurred or was necessary, the AE should be considered serious.

   Hospitalization for elective treatment of a pre-existing condition that did not worsen from baseline is not considered an AE.

d. Results in disability/incapacity, or

   Note: The term disability means a substantial disruption of a person's ability to conduct normal life functions. This definition is not intended to include experiences of relatively minor medical significance such as uncomplicated headache, nausea, vomiting, diarrhea, influenza, and accidental trauma (e.g., sprained ankle) which may interfere or prevent everyday life functions but do not constitute a substantial disruption.

e. Is a congenital anomaly/birth defect

f. Medical or scientific judgment should be exercised in deciding whether reporting is appropriate in other situations, such as important medical events that may not be immediately life-threatening or result in death or hospitalization but may jeopardize the subject or may require medical or surgical intervention to prevent one of the other outcomes listed in the above definition. These should also be considered serious. Examples of such events are invasive or malignant cancers, intensive treatment in an emergency room or at home for allergic bronchospasm, blood dyscrasias or convulsions that do not result in hospitalization, or development of drug dependency or drug abuse.

g. Is associated with liver injury **and** impaired liver function defined as:

- ALT ≥ 3xULN, and
- total bilirubin ≥ 2xULN or INR > 1.5.

NOTES:

Bilirubin fractionation should be performed if testing is available. If fractionation is unavailable, urinary bilirubin is to be measured via dipstick (a measurement of direct bilirubin, which would suggest liver injury).

INR measurement is not required; if measured, the threshold value stated will not apply to patients receiving anticoagulants. If INR measurement is obtained, the value is to be recorded on the SAE form.

- Refer to Section 13 for the required liver chemistry follow-up instructions.

12.3. Oral Adverse Events

All subjects with an oral adverse event suspicious of osteonecrosis of the jaw (ONJ) should be examined by a dentist or other qualified oral specialist (e.g., oral surgeon). Oral AEs reported as ONJ or identified by GSK as potentially representing ONJ will be reviewed by an independent adjudication panel of experts to determine whether the pre-defined criteria for ONJ are met. GSK will request the investigating site to provide all available source documents surrounding that event to be reviewed by the blinded adjudication committee. If an event is adjudicated positively for ONJ, the investigator will be notified of the adjudication decision; and the event will be upgraded to an SAE and reported to the regulatory agencies and study investigators in an expedited manner.

12.4. Atypical Femoral Fracture

Femoral fracture adverse events will be reviewed by an independent adjudication committee for suspicion of being an atypical femoral fracture (AFF). AFF are defined as subtrochanteric or proximal diaphyseal fractures that occur with little to no trauma and are characterized by specific radiographic findings.

Screening will consist of regular review of AE and SAE reports, and by using a predefined list of related Medical Dictionary for Regulatory Activities (MedDRA) preferred terms. For identified cases of possible AFF, GSK will request the investigating site provide all available source documents surrounding that event and completed an AFF

adjudication questionnaire. An independent adjudication panel of blinded AFF experts will determine if diagnostic criteria are met. If an event is adjudicated positively for AFF the investigator will be notified of the adjudication decision; and the events will be upgraded to an SAE if not already reported as such, and reported to the regulatory agencies and study investigators in an expedited manner.

12.5. Method of Detecting AEs and SAEs

Care will be taken not to introduce bias when detecting AEs and/or SAEs. Open-ended and non-leading verbal questioning of the subject is the preferred method to inquire about AE occurrence. Appropriate questions include:

- "How are you feeling?"
- "Have you had any (other) medical problems since your last visit/contact?"
- "Have you taken any new medicines, other than those provided in this study, since your last visit/contact?"

12.6. Recording of AEs and SAEs

When an AE/SAE occurs, it is the responsibility of the investigator to review all documentation (e.g., hospital progress notes, laboratory, and diagnostics reports) relative to the event. The investigator will then record all relevant information regarding an AE/SAE in the appropriate data collection tool.

It is not acceptable for the investigator to send photocopies of the subject's medical records to GSK in lieu of completion of the GSK, AE/SAE data collection tool. However, there may be instances when copies of medical records for certain cases are requested by GSK. In this instance, all subject identifiers, with the exception of the subject number, will be blinded on the copies of the medical records prior to submission to GSK.

The investigator will attempt to establish a diagnosis of the event based on signs, symptoms, and/or other clinical information. In such cases, the diagnosis will be documented as the AE/SAE and not the individual signs/symptoms.

12.7. Evaluating AEs and SAEs

12.7.1. Assessment of Intensity

The investigator will make an assessment of intensity for each AE and SAE reported during the study and will assign it to one of the following categories:

Mild: An event that is easily tolerated by the subject, causing minimal discomfort and not interfering with everyday activities.

Moderate: An event that is sufficiently discomforting to interfere with normal everyday activities.

Severe: An event that prevents normal everyday activities.

An AE that is assessed as severe will not be confused with an SAE. Severity is a category utilized for rating the intensity of an event; and both AEs and SAEs can be assessed as severe. An event is defined as 'serious' when it meets at least one of the pre-defined outcomes as described in the definition of an SAE.

12.7.2. Assessment of Causality

The investigator is obligated to assess the relationship between investigational product and the occurrence of each AE/SAE. A "reasonable possibility" is meant to convey that there are facts/evidence or arguments to suggest a causal relationship, rather than a relationship cannot be ruled out. The investigator will use clinical judgment to determine the relationship. Alternative causes, such as natural history of the underlying diseases, concomitant therapy, other risk factors, and the temporal relationship of the event to the investigational product will be considered and investigated. The investigator will also consult the Investigator Brochure (IB) and/or Product Information, for marketed products, in the determination of his/her assessment.

There may be situations when an SAE has occurred and the investigator has minimal information to include in the initial report to GSK. However, **it is very important that the investigator always make an assessment of causality for every event prior to the initial transmission of the SAE data to GSK.** The investigator may change his/her opinion of causality in light of follow-up information, amending the SAE data collection tool accordingly. The causality assessment is one of the criteria used when determining regulatory reporting requirements.

12.8. Follow-up of AEs and SAEs

After the initial AE/SAE report, the investigator is required to proactively follow each subject at subsequent visits/contacts. All AEs and SAEs will be followed until resolution, until the condition stabilizes, until the event is otherwise explained, or until the subject is lost to follow-up.

The investigator is obligated to perform or arrange for the conduct of supplemental measurements and/or evaluations as may be indicated or as requested by GSK to elucidate as fully as possible the nature and/or causality of the AE or SAE. The investigator is obligated to assist. This may include additional laboratory tests or investigations, histopathological examinations or consultation with other health care professionals. If a subject dies during participation in the study or during a recognized follow-up period, the investigator will provide GSK with a copy of any post-mortem findings, including histopathology.

New or updated information will be recorded in the originally completed data collection tool. The investigator will submit any updated SAE data to GSK within the designated reporting time frames.

12.9. Prompt Reporting of SAEs to GSK

Once the investigator determines that an event meets the protocol definition of an SAE, the SAE will be reported to GSK **within 24 hours**. Any follow-up information on a previously reported SAE will also be reported to GSK within 24 hours.

If the investigator does not have all information regarding an SAE, he/she will not wait to receive additional information before notifying GSK of the event and completing the appropriate data collection tool. The investigator will always provide an assessment of causality at the time of the initial report as described in Section 12.7.2, Assessment of Causality.

The primary mechanism for reporting SAEs to GSK will be the electronic data collection tool (e.g., InForm system). If the electronic system is unavailable for greater than 24 hours, the site will use the paper SAE data collection tool and fax it to the GSK Medical Monitor or protocol contact. Then the site will enter the serious adverse event data into the electronic system as soon as it becomes available.

After the study is completed at a given site, the electronic data collection tool (e.g., InForm system) will be taken off-line to prevent the entry of new data or changes to existing data. If a site receives a report of a new SAE from a study participant or receives updated data on a previously reported SAE after the electronic data collection tool has been taken off-line, the site can report this information on a paper SAE form or to their GSK protocol contact by telephone.

GSK contacts for SAE receipt can be found at the beginning of this protocol on the Sponsor/Medical Monitor Contact Information page.

12.10. Regulatory Reporting Requirements For SAEs

Prompt notification of SAEs by the investigator to GSK is essential so that legal obligations and ethical responsibilities towards the safety of subjects are met.

GSK has a legal responsibility to notify both the local regulatory authority and other regulatory agencies about the safety of a product under clinical investigation. GSK will comply with country specific regulatory requirements relating to safety reporting to regulatory authorities, IRBs/IECs and investigators.

Investigator safety reports are prepared for suspected unexpected serious adverse reactions according to local regulatory requirements and GSK policy and are forwarded to investigators as necessary. An investigator who receives an investigator safety report describing an SAE(s) or other specific safety information (e.g., summary or listing of SAEs) from GSK will file it with the IB and will notify the IRB/IEC, if appropriate according to local requirements.

13. LIVER CHEMISTRY FOLLOW-UP PROCEDURES

The procedures listed below are to be followed if a subject has ALT, bilirubin and/or INR elevations that meet the definition of an SAE (as defined in Section 12.2):

- Notify the GSK medical monitor within 24 hours of learning of the abnormality to confirm follow-up.
- Complete the liver event case report forms.
- Upon completion of the safety follow-up permanently withdraw the subject from the study and do not rechallenge with investigational product.
- Make every reasonable attempt to have subjects return to the clinic within 24 hours for repeat liver chemistries, additional testing, and close monitoring (with specialist or hepatology consultation recommended).
- Monitor subjects <u>twice weekly</u> until liver chemistries (ALT, AST, alkaline phosphatase, bilirubin) resolve, stabilize or return to within baseline values.
- Obtain viral hepatitis serology including:
  - Hepatitis A IgM antibody.
  - Hepatitis B surface antigen and Hepatitis B Core Antibody (IgM).
  - Hepatitis C RNA.
  - Cytomegalovirus IgM antibody.
  - Epstein-Barr viral capsid antigen IgM antibody (or if unavailable, obtain heterophile antibody or monospot testing).
  - Hepatitis E IgM antibody
- Serum creatine phosphokinase (CPK) and lactate dehydrogenase (LDH).
- Assess eosinophilia
- Record the appearance or worsening of clinical symptoms of hepatitis (fatigue, nausea, vomiting, right upper quadrant pain or tenderness, fever, rash or eosinophilia) on the AE CRF.
- Record use of concomitant medications, acetaminophen, herbal remedies, other over the counter medications, or putative hepatotoxins on the Concomitant Medications CRF.
- Record alcohol use on the Liver Events CRF.
- Anti-nuclear antibody, anti-smooth muscle antibody, and Type 1 anti-liver kidney microsomal antibodies.
- Liver imaging (ultrasound, magnetic resonance, or computerized tomography) to evaluate liver disease.
- The Liver Imaging and/or Liver Biopsy CRFs are also to be completed if these tests are performed.

14. STUDY CONDUCT CONSIDERATIONS

14.1. Posting of Information on Clinicaltrials.gov

Study information from this protocol will be posted on clinicaltrials.gov before enrollment of subjects begins.

14.2. Regulatory and Ethical Considerations, Including the Informed Consent Process

GSK will obtain favorable opinion/approval to conduct the study from the appropriate regulatory agency in accordance with any applicable country-specific regulatory requirements prior to a site initiating the study in that country.

The study will be conducted in accordance with all applicable regulatory requirements.

The study will also be conducted in accordance with "good clinical practice" (GCP), all applicable subject privacy requirements, and, the guiding principles of the 2008 Declaration of Helsinki. This includes, but is not limited to, the following:

- IRB/IEC review and favorable opinion/approval to conduct the study and of any subsequent relevant amended documents.
- Written informed consent (and any amendments) to be obtained for each subject before participation in the study.
- Investigator reporting requirements (e.g., reporting of AEs/SAEs/protocol deviations to IRB/IEC).

14.2.1. Urgent Safety Measures

If an event occurs that is related to the conduct of the study or the development of the investigational product, and this new event is likely to affect the safety of subjects, the sponsor and the investigator will take appropriate urgent safety measures to protect subjects against any immediate hazard.

The sponsor will work with the investigator to ensure the IEC/IRB is notified.

14.3. Quality Control (Study Monitoring)

In accordance with applicable regulations including GCP, and GSK procedures, GSK monitors will contact the site prior to the start of the study to review with the site staff the protocol, study requirements, and their responsibilities to satisfy regulatory, ethical, and GSK requirements. When reviewing data collection procedures, the discussion will also include identification, agreement and documentation of data items for which the CRF will serve as the source document.

GSK will monitor the study and site activity to verify that the:

- Data are authentic, accurate, and complete.

- Safety and rights of subjects are being protected.
- Study is conducted in accordance with the currently approved protocol and any other study agreements, GCP, and all applicable regulatory requirements.
- The investigator and the head of the medical institution (where applicable) agrees to allow the monitor direct access to all relevant documents.

14.4. Quality Assurance

To ensure compliance with GCP and all applicable regulatory requirements, GSK may conduct a quality assurance audit. Regulatory agencies may also conduct a regulatory inspection of this study. Such audits/inspections can occur at any time during or after completion of the study. If an audit or inspection occurs, the investigator and institution agree to allow the auditor/inspector direct access to all relevant documents and to allocate his/her time and the time of his/her staff to the auditor/inspector to discuss findings and any relevant issues.

14.5. Study and Site Closure

Upon completion or premature discontinuation of the study, the monitor will conduct site closure activities with the investigator or site staff, as appropriate, in accordance with applicable regulations including GCP, and GSK procedures.

In addition, GSK reserves the right to temporarily suspend or prematurely discontinue this study at any time for reasons including, but not limited to, safety or ethical issues or severe non-compliance. For multicenter studies, this can occur at one or more or at all sites. If GSK determines such action is needed, GSK will discuss this with the investigator or the head of the medical institution (where applicable), including the reasons for taking such action. When feasible, GSK will provide advance notification to the investigator or the head of the medical institution, where applicable, of the impending action prior to it taking effect.

If the study is suspended or prematurely discontinued for safety reasons, GSK will promptly inform investigators or the head of the medical institution (where applicable) and the regulatory authorities of the suspension or premature discontinuation of the study and the reason(s) for the action. If required by applicable regulations, the investigator or the head of the medical institution (where applicable) must inform the IRB/IEC promptly and provide the reason for the suspension or premature discontinuation.

14.6. Records Retention

Following closure of the study, the investigator or the head of the medical institution (where applicable) must maintain all site study records, except for those required by local regulations to be maintained by someone else, in a safe and secure location. The records must be maintained to allow easy and timely retrieval, when needed (e.g., audit or inspection), and, whenever feasible, to allow any subsequent review of data in conjunction with assessment of the facility, supporting systems, and staff. Where permitted by local laws/regulations or institutional policy, some or all of these records

can be maintained in a format other than hard copy (e.g., microfiche, scanned, electronic); however, caution needs to be exercised before such action is taken. The investigator must assure that all reproductions are legible and are a true and accurate copy of the original, and meet accessibility and retrieval standards, including re-generating a hard copy, if required. Furthermore, the investigator must ensure there is an acceptable back-up of these reproductions and that an acceptable quality control process exists for making these reproductions.

GSK will inform the investigator of the time period for retaining these records to comply with all applicable regulatory requirements. The minimum retention time will meet the strictest standard applicable to that site for the study, as dictated by any institutional requirements or local laws or regulations, or GSK standards/procedures; otherwise, the retention period will default to 15 years.

The investigator must notify GSK of any changes in the archival arrangements, including, but not limited to, archival at an off-site facility or transfer of ownership of the records in the event the investigator leaves the site.

14.7. Provision of Study Results to Investigators, Posting to the Clinical Trials Register and Publication

Where required by applicable regulatory requirements, an investigator signatory will be identified for the approval of the clinical study report. The investigator will be provided reasonable access to statistical tables, figures, and relevant reports and will have the opportunity to review the complete study results at a GSK site or other mutually-agreeable location.

GSK will also provide the investigator with the full summary of the study results. The investigator is encouraged to share the summary results with the study subjects, as appropriate.

GSK will provide the investigator with the randomization codes for their site only after completion of the full statistical analysis.

The results summary will be posted to the Clinical Study Register at the time of the first regulatory approval or within 12 months of any decision to terminate development. In addition, a manuscript will be submitted to a peer reviewed journal for publication no later than 12 months after the first approval or any decision to terminate development. When manuscript publication in a peer reviewed journal is not feasible, further study information will be posted to the GSK Clinical Study Register to supplement the results summary.

14.8. Data Management

GSK Data Management will identify and implement the most effective data acquisition and management strategy for each clinical trial protocol and deliver datasets which support the protocol objectives. Subject data will be entered into GSK defined CRFs and combined with data provided from other sources (e.g., diary data, laboratory data) in a validated data system. Subject initials will not be transmitted to GSK for inclusion in the

datasets. Clinical data management will be performed in accordance with applicable GSK standards and data cleaning procedures with the objective of removing errors and inconsistencies in the data which would otherwise impact on the analysis and reporting objectives, or the credibility of the Clinical Study Report. Adverse events and concomitant medications terms will be coded using validated dictionaries. Original CRFs will be retained by GSK, while the investigator will retain a copy.

14.9. Osteonecrosis of the Jaw Adjudication Committee

As part of enhanced and ongoing safety monitoring of osteonecrosis of the jaw, GSK, in cooperation with Amgen, has established the Osteonecrosis of the Jaw Adjudication Committee (ONJAC), which will independently adjudicate all potential ONJ events identified during denosumab clinical trials. The external committee is charged specifically with providing a review and adjudication of all investigator-reported conditions possibly related to or suspicious for ONJ, according to GSK/Amgen's pre-established event definition.

GSK recognizes that subjects treated with intravenous and oral bisphosphonates are at an increased risk for developing ONJ, although the overall incidence of this condition is not clearly defined for all marketed products. Some subjects have also received bisphosphonates prior to enrollment on GSK clinical trials.

ONJ has been observed in patients receiving denosumab for advanced cancer and rarely in patients with PMO. Hence, GSK seeks to monitor all subjects in the denosumab development program for occurrence of ONJ and has established ONJAC experts to examine potential cases during the course of the study, while remaining blinded to the subject treatment assignment.

14.10. Atypical Femoral Fracture Adjudication Committee (AFFAC)

Cases of atypical femoral fracture have been reported in the osteoporosis literature in association with long-term bisphosphonate use. Some case series have reported a possible association between atypical femoral fracture and long-term alendronate therapy, with low bone turnover as the suggested etiology [Lenart, 2008; Odvina, 2005; Odvina 2010], while others have not [Abrahamsen, 2009]. Atypical femoral fracture has been confirmed in an ongoing clinical trial 20060289 with denosumab. All events reported as atypical femoral fractures, or those coded to pre-specified terms potentially indicative of atypical femoral fractures, will be reviewed by an independent adjudication committee.

15. REFERENCES

Abrahamsen B, Eiken P, Eastell R. 2009. Subtrochanteric and diaphyseal femural fractures in patients treated with alendronate: a register-based national cohort study. J Bone Miner Res 24, 1095-1102.

Amgen Protocol Number 20050146. An open-label, randomized, single-dose, parallel group study in healthy volunteers to assess the bioequivalence of denosumab after subcutaneous (SC) administrations with a pre-filled syringe (PFS) versus a graduated syringe. May, 2006

Amgen Protocol Number 20060446. An open-label, randomized, single-dose, parallel group study in healthy volunteers to assess the bioequivalence of denosumab 120mg subcutaneous dose when administered as two injections of 60mg/mL or as a single injection of 70mg/mL. July, 2007

Anderson DM, Maraskovsky E, Billingsley WL et al. 1997. A homologue of the TNF receptor and its ligand enhance T cell growth and dendritic function. Nature 390, 175-179

Bucay N, Sarosi I, Dunstan CR et al. 1998. Osteoprotegerin-deficient mice develop early onset osteoporosis and arterial calcification. Genes Develop 12, 1260-1268

Burgess TL, Qian Y, Kaufman S. 1999. The ligand for osteoprotegerin (OPGL) directly activates mature osteoclasts. J Cell Biol 145, 527-538

Burr D, Martin, RB. 1989. Errors in bone remodeling: towards a unified theory of metabolic bone disease. Am J Anat 186, 186-216

Denosumab (AMG 162) Investigator's Brochure, Edition 2.0, 23 August 2013

Eghbali-Fatourechi G, Khosla S, Sanyal A, Boyle WJ, Lacey DL, Riggs BL. 2003. Role of RANK ligand in mediating increased bone resorption in early postmenopausal women. J Clin Invest 111, 1221-1230.

Elliott R, Kostenuik P, Chen C, et al. 2006. Denosumab is a selective inhibitor of human receptor activator of NF-κβ ligand that blocks osteoclast formation in vitro and in vivo. Eur J Cancer Suppl 4, 62

Kong Y-Y, Feige U, Sarosi I, et al. 1999. Activated T cells regulate bone loss and joint destruction in adjuvant arthritis through osteoprotegerin ligand. Nature 402, 304-309.

Kostenuik PJ, Capparelli C, Morony S, et al. 2001. OPG and PTH-(1-34) have additive effects on bone density and mechanical strength in osteopenic ovariectomized rats. Endocrinology 142, 4295-4304

Lacey DL, Timms E, Tan H-L, et al. 1998. Osteoprotegerin ligand is a cytokine that regulates osteoclast differentiation and activation. Cell 93, 165-176

Lau EMC, Chan HHL, et al. 1996. Body composition and bone mineral density of Chinese women with vertebral fracture. Bone 19,657-662

Lenart BA, Lorich DG, Lane JM. 2008. Atypical fractures of the femoral diaphysis in postmenopausal women taking alendronate. N Engl J Med 358, 1304-1306.

Odvina C, Zerwekh JE, Rao S, et al. 2005. Severely suppressed bone turnover: a potential complication of alendronate therapy. J Clin Endocrinol Metab 90, 1294-1301.

Odvina C, Zerwekh JE, Rao S, et al. 2010. Unusual mid-shaft fractures during long term bisphosphonate therapy. Clin Endocrinol 72, 161-168.

Simonet WS, Lacey DL, Dunstan CR et al. 1997. Osteoprotegerin: A novel secreted protein involved in the regulation of bone density. Cell 89, 309-319

Yasuda H, Shima N, Nakagawa N, et al. 1998. Osteoclast differentiation factor in ligand for osteoprotegerin/osteoclastogenesis-inhibitory factor and is identical to TRANCE/RANKL. Proc Natl Acad Sci USA 95, 3597-360.

APPENDICES

Appendix 1: Protocol Amendment Changes

AMENDMENT 2

Where the Amendment Applies

This amendment applies to all sites.

Summary of Amendment Changes with Rationale

The primary intent of this amendment is to revise the calcium supplementation from "at least 1000 mg" to "at least 600 mg" to ensure the dose is equal to the Denosumab China postmenopausal osteoporosis phase III study in China. The information about atypical femoral fracture is added in this protocol because of the risk and management plan updated. Modify the eligibility criteria by adding the syphilis test and chest X-ray test per investigator's suggestion. PK sample analysis will be performed by Wuxi AppTec instead of PPD, Virgina, USA. Additional changes are listed as below.

- Update time and events table, including specify the Day and Week, add chest X-ray exam and syphilis test at screening, clarify urine pregnancy test will be used for women in Day -1, add urine pregnancy test for women during the study period, add physical examinations, ECG and clinical chemistry, hematology and urinalysis on Day -1.
- Delete redundancy description of prior diagnosis of certain diseases in exclusion criteria to avoid ambiguity.
- Specify the collection of blood sample in study assessments and procedures per investigator's suggestion.
- Specify the volume of blood sampling and the detecting methods of s-CTX1 and denosumab antibody assay per investigator's suggestion.
- Modify the requirement of collection screen failure information because of GSK's new policy.
- Clarify the single-blind is blinded to subjects only due to typographical error.

<u>Change 1</u>

Section 1.1.3.1 Safety Profile

PREVIOUS TEXT

- Denosumab did not result in increased incidence of hypersensitivity reactions or potential clinical sequelae of hypersensitivity.

REVISED TEXT

- Denosumab did not result in increased incidence of hypersensitivity reactions or potential clinical sequelae of hypersensitivity **in clinical trials**. **In the post-marketing setting, rare events of drug-related hypersensitivity, including anaphylaxis have been reported in patients receiving denosumab. Denosumab is contraindicated in individuals with clinically significant hypersensitivity.**

Rationale: Clarify the description of hypersensitivity in the safety profile.

Change 2

Section 1.3 Dose Rationale

PREVIOUS TEXT

- Doses of 60 mg and 120 mg represent the doses proposed for approval: the 60 mg dose has been submitted for approval for the treatment of osteoporosis in postmenopausal women and the treatment of bone loss associated with hormone ablation in men with prostate cancer and in women with breast cancer; the 120 mg dose is the clinical dose currently under evaluation in patients with advanced cancer (SRE);

REVISED TEXT

- Doses of 60 mg and 120 mg represent the doses proposed for approval **in China**: the 60 mg dose has been ~~submitted for approval~~ approved for the treatment of osteoporosis in postmenopausal women and the treatment of bone loss associated with hormone ablation in men with prostate cancer and in women with breast cancer **in many other countries and regions**; the 120 mg dose **has been approved for prevention of skeletal related events (SRE) in patients with bone metastases from solid tumors** ~~is the clinical dose currently under evaluation in patients with advanced cancer (SRE)~~;

Rationale: Clarify denosumab 60 mg and 120 mg are applied for approval in China.

Change 3

Section 1.4 Summary of Risk Management, Paragraph 1

PREVIOUS TEXT

Denosumab has been generally well tolerated in all clinical studies conducted to date. This study represents the first trial involving subjects in China.

REVISED TEXT

Denosumab has been generally well tolerated in all clinical studies conducted to date. ~~This study represents the first trial involving subjects in China.~~

Rationale: Update the information because two phase III trials about denosumab are ongoing in China.

Change 4

Section 1.4 Summary of Risk Management

PREVIOUS TEXT

Risk	Impact on eligibility criteria	Monitoring
Osteonecrosis of Jaw (ONJ)	Exclusion criteria:<br><br>- Prior history or current evidence of osteomyelitis or ONJ<br><br>- Active dental or jaw condition that requires oral surgery<br><br>- Planned invasive dental procedure for the course of the study<br><br>- Non-healed dental or oral surgery	Monitor oral AE events. All subjects with an oral AE suspicious of ONJ should be examined by a dentist or other qualified oral specialist. Oral AEs reported as or suspected to be ONJ will be reviewed by an independent adjudication panel of experts.
Transient hypocalcemia (seen in some Dmab-treated subjects)	Exclusion criterion:<br><br>Abnormal serum calcium at screen	Monitor serum calcium levels<br><br>Supplementation with calcium and vitamin D during the study
Immunogenicity (theoretical risk of MAbs)	None	Collection of antibody samples in ongoing studies

REVISED TEXT

Risk	Impact on eligibility criteria	Monitoring
Osteonecrosis of the Jaw (ONJ)	Exclusion criteria:<br><br>- Prior history or current evidence of osteomyelitis or ONJ<br><br>- Active dental or jaw condition that requires oral surgery<br><br>- Planned invasive dental procedure for the course of the study<br><br>- Non-healed dental or oral surgery	Monitor oral AE events. All subjects with an oral AE suspicious of ONJ should be examined by a dentist or other qualified oral specialist. Oral AEs reported as or suspected to be ONJ will be reviewed by an independent adjudication panel of experts.
**Atypical Femoral Fracture (AFF)**	**None**	**Monitor femoral fracture events. AFF reports will be reviewed by an independent adjudication panel of experts.**
Transient hypocalcemia (seen in some Dmab-treated subjects)	Exclusion criterion:<br><br>Abnormal serum calcium at screen	Monitor serum calcium levels<br><br>Supplementation with calcium and vitamin D during the study
Immunogenicity (theoretical risk of MAbs)	None	Collection of antibody samples in ongoing studies
**Hypersensitivity**	**Exclusion of subjects with a history of sensitivity to any of the study medications, especially known sensitivity to mammalian-derived drug preparations, or components thereof or a history of drug or other allergy that, in the opinion of the investigator or GSK Medical Monitor,**	**Monitoring post-dosing**

Risk	Impact on eligibility criteria	Monitoring
	**contraindicates their participation**	

Rationale: Add information of atypical femoral fracture and hypersensitivity to update the risk management.

Change 5

Section 4.1.2 Treatment, Paragraph 4

PREVIOUS TEXT

All subjects will be provided with daily vitamin D (at least 400 IU but < 1000 IU/day) and calcium (at least 1000 mg) supplementation for the duration of the study.

REVISED TEXT

All subjects will be provided with daily vitamin D (at least 400 IU but < 1000 IU/day) and calcium (at least ~~1000~~ **600** mg) supplementation for the duration of the study.

Rationale: Revise the dose of calcium supplementation to ensure it is aligned with that in Denosumb China postmenopausal osteoporosis phase III study.

Change 6

Section 4.5 Time and Events Table

PREVIOUS TEXT

**Day:**	Screening	-1	1	2	3	4	5	6	8	11											Follow-up (via Phone)
**Week:**											3	4	5	7	9	11	13	15	17	19	26
**Visit Window** (relative to Day 1)	-21 to -1 days	NA									±2 days										
**Admission to Unit**		X																			
**Informed Consent**	X																				
**Inclusion/Exclusion**	X																				
**Demographics**	X																				
**Physical Examination**	X																			X	
**Medical history**	X																				
**12-lead ECG**	X[1]																			X	
**Vital signs(BP and HR)**	X	X	X[2]	X	X	X	X	X	X	X	X	X	X	X	X	X	X	X	X	X	
**Height**	X																				
**Weight**	X																			X	
**Urine drug/alc. screen**	X	X																			
**<u>Serum</u> β-hCG pregnancy test (women)**	X	X																			
**HIV, Hep B and Hep C**	X																				
**Hema/Chem/Urinalysis**	X								X[3]				X				X			X	
**AE assessment**	X[4]	X[4]	X	X	X	X	X	X	X	X	X	X	X	X	X	X	X	X	X	X	X
**Pregnancy assessment phone call**																					X
**Conmed Review**			X	X	X	X	X	X	X	X	X	X	X	X	X	X	X	X	X	X	
**PK blood sample**			X[5]	X[5]	X[5]	X	X	X	X	X	X	X	X	X	X	X	X	X	X	X	
**PD (CTX1) sample**			X[2]	X	X	X	X	X	X	X	X	X	X	X	X	X	X	X	X	X	
**Antibody sample**			X[2]									X			X					X	

Day:	Screening	-1	1	2	3	4	5	6	8	11											Follow-up (via Phone)
Week:											3	4	5	7	9	11	13	15	17	19	26
**Visit Window** (relative to Day 1)	-21 to -1 days	NA									±2 days										
**Dosing**			X																		
**Discharge**				X																	
**Outpatient visit**	X				X	X	X	X	X	X	X	X	X	X	X	X	X	X	X	X	

Time and Events Table (Continued)

1. Triplicate ECG at Screening to confirm eligibility. Single ECG at other time points.
2. Assessments performed prior to dosing.
3. Only serum calcium is required on Day 8.
4. Only SAEs related to study participation will be recorded from the signing of consent to study drug administration.
5. PK blood samples will be drawn pre-dose, 1hr, 4 hrs, 8 hrs, 12 hrs, 24 hrs (Day 2), and 48 hrs (Day 3) post-dose.

REVISED TEXT

Day:	Screening	d-1	d1	d2	d3	d4	d5	d6	d8	d11	**d21**	**d28**	**d35**	**d49**	**d63**	**d77**	**d91**	**d105**	**d119**	**d133/ early withdrawal**	**d182** Follow-up (via Phone)
Week:									w2		w3	w4	w5	w7	w9	w11	w13	w15	w17	w19	w26
**Visit Window** (relative to Day 1)	-21 to -1 days	NA									±2 days										
**Admission to Unit**		X																			
**Informed Consent**	X																				
**Inclusion/Exclusion**	X	**X**																			
**Demographics**	X																				
**Physical Examination**	X	**X**																		X	
**Medical history**	X																				
**12-lead ECG**	X[1]	**X**																		X	

**Day:**	Screening	d-1	d1	d2	d3	d4	d5	d6	d8	d11	**d21**	**d28**	**d35**	**d49**	**d63**	**d77**	**d91**	**d105**	**d119**	**d133/ early withdrawal**	**d182** Follow-up (via Phone)
**Week:**									w2		w3	w4	w5	w7	w9	w11	w13	w15	w17	w19	w26
**Visit Window** (relative to Day 1)	-21 to -1 days	NA									±2 days										
**Chest X Ray Exam**		X																			
**Vital signs(BP and HR)**	X	X	X[2]	X	X	X	X	X	X	X	X	X	X	X	X	X	X	X	X	X	
**Height**	X																				
**Weight**	X																			X	
**Urine drug/alc. screen**	X	X																			
**Urine pregnancy test (women)**		**X**											**X**				**X**			**X**	
**Serum β-hCG pregnancy test (women)**	X																				
**HIV, Hep B and Hep C, Syphilis screen**	X																				
**Hema/Chem/Urinalysis**	X	**X[6]**							X[3]				X				X			X	
**AE assessment**	X[4]	X[4]	X	X	X	X	X	X	X	X	X	X	X	X	X	X	X	X	X	X	X
**Pregnancy assessment phone call**																					X
**Conmed Review**			X	X	X	X	X	X	X	X	X	X	X	X	X	X	X	X	X	X	
**PK blood sample**			X[5]	X[5]	X[5]	X	X	X	X	X	X	X	X	X	X	X	X	X	X	X	
**PD (s-CTX1) sample**			X[2]	X	X	X	X	X	X	X	X	X	X	X	X	X	X	X	X	X	
**Antibody sample**			X[2]									X			X					X	
**Dosing**			X																		
**Discharge from Unit**				X																	
**Outpatient visit**	X				X	X	X	X	X	X	X	X	X	X	X	X	X	X	X	X	

1. Triplicate ECG at Screening to confirm eligibility. Single ECG at other time points.
2. Assessments performed prior to dosing.
3. Only serum calcium is required on Day 8.
4. Only SAEs related to study participation will be recorded from the signing of consent to study drug administration.
5. PK blood samples will be drawn pre-dose, 1hr, 4 hrs, 8 hrs, 12 hrs, 24 hrs (Day 2), and 48 hrs (Day 3) post-dose.
6. **If Screening visit is within 14 days prior to Day -1, then Hema/Chem/Urialysis tests at Day -1 can be exemption.**

Rationale: Specify the Day and Week, specify the items of early withdrawal, add chest X- ray exam and syphilis test at screening per investigator's suggestion, clarify urine pregnancy test will be used for women in Day -1 because the serum hCG test result is unavailable from central lab within that day, add urine pregnancy tests for women during the study period to identify the pregnancy status of female subjects. Because of typo graphic error, add physical examinations, ECG and clinical chemistry, hematology and urinalysis on Day -1to make it align with the description in Section 4.1.2.

Change 7

Section 5.2.2 Exclusion Criteria

PREVIOUS TEXT

No previous text.

REVISED TEXT

Add

9. **A positive test for syphilis at Screening.**

Rationale: According to investigator's suggestion to exclude subjects who are positive for syphilis.

PREVIOUS TEXT

9. Abnormal serum calcium: current hypocalcemia or hypercalcemia. Albumin-adjusted serum calcium levels must be within the normal range of the local laboratory.

REVISED TEXT

10. Abnormal serum calcium: current hypocalcemia or hypercalcemia. Albumin-adjusted serum calcium levels must be within the normal range of the ~~local~~ **central** laboratory.

Rationale: Typo graphic error.

PREVIOUS TEXT

15. Pregnant females as determined by positive serum hCG test at screening or prior to dosing (visit -1).

REVISED TEXT

16. Pregnant females as determined by positive serum hCG test at screening or **urine hCG test** prior to dosing (~~visit~~ **day** -1).

Rationale: Clarify the pregnancy test method and procedure as the central lab cannot return the serum hCG test result at the day of testing on day -1.

PREVIOUS TEXT

No previous text.

REVISED TEXT

Add

17. **A chest X-ray or computed tomography (CT) scan that reveals evidence of clinical significant abnormalities e.g., tuberculosis. A chest X-ray must be taken at Day-1 if a chest X-ray or CT scan is not available within 6 months prior to that day.**

Rationale: According to investigator's suggestion to exclude clinical significant abnormalities.

PREVIOUS TEXT

18. Prior diagnosis of bone disease, or any condition that will effect bone metabolism such as, but not limited to:
    - Osteoporosis
    - Osteogenesis imperfecta
    - Hyperparathyroidism
    - Hyperthyroidism
    - Hypothyroidism (acceptable if on stable thyroid replacement therapy greater than 6 months and serum TSH values are within normal limits)
    - Osteomalacia
    - Rheumatoid arthritis
    - Psoriatic arthritis
    - Ankylosing spondylitis
    - Current flare-up of osteoarthritis and/or gout
    - Active malignancy
    - Renal disease (defined as GFR < 45 mL/min)
    - Paget's disease of the bone
    - Recent bone fracture (within 6 months)
    - Malabsorption syndrome

REVISED TEXT

Delete

20. ~~Prior diagnosis of bone disease, or any condition that will effect bone metabolism such as, but not limited to:~~
    - ~~Osteoporosis~~
    - ~~Osteogenesis imperfecta~~
    - ~~Hyperparathyroidism~~
    - ~~Hyperthyroidism~~
    - ~~Hypothyroidism (acceptable if on stable thyroid replacement therapy greater than 6 months and serum TSH values are within normal limits)~~
    - ~~Osteomalacia~~
    - ~~Rheumatoid arthritis~~
    - ~~Psoriatic arthritis~~
    - ~~Ankylosing spondylitis~~
    - ~~Current flare-up of osteoarthritis and/or gout~~
    - ~~Active malignancy~~
    - ~~Renal disease (defined as GFR < 45 mL/min)~~
    - ~~Paget's disease of the bone~~
    - ~~Recent bone fracture (within 6 months)~~
    - ~~Malabsorption syndrome~~

Rationale: Delete the redundant description of certain diseases history because the definition of healthy is already explained in inclusion criteria 3.

Change 8

Section 7 STUDY ASSESSMENTS AND PROCEDURES, Paragraph 2

PREVIOUS TEXT

The timing and number of planned study assessments, including safety, pharmacokinetic, pharmacodynamic/biomarker or others assessments may be altered during the course of the study based on newly available data to ensure appropriate monitoring. The change in timing or addition of time points for any planned study assessments must be approved and documented by GSK, but this will not constitute a protocol amendment. The IRB/IEC will be informed of any safety issues that require alteration of the safety monitoring scheme. No more than 500 mL of blood will be collected over the duration of the study, including any extra assessments that may be required.

REVISED TEXT

The timing and number of planned study assessments, including safety, pharmacokinetic, pharmacodynamic/biomarker or others assessments may be altered during the course of the study based on newly available data to ensure appropriate monitoring. The change in

timing or addition of time points for any planned study assessments must be approved and documented by GSK, but this will not constitute a protocol amendment. The IRB/IEC will be informed of any safety issues that require alteration of the safety monitoring scheme. **There will be 22 time points for PK blood sample collection and approximately 3.5 mL of blood will be collected each time. There will be 18 time points for PD blood sample collection and approximately 3.5 mL of blood will be collected each time. Haematological and chemical assessment will be tested for 5 times, approximately 6.5 mL of blood will be collected for each test. Virological assessment will be tested at screening and approximately 3 mL of blood will be collected. Blood pregnancy test will be tested at screening, approximately 1.5 mL of blood will be collected. Denosumab antibody will be tested for 4 times and 7 mL of blood will be collected each time.** ~~No more than~~ **Approximately 200 mL** of blood will be collected over the duration of the study~~, including any extra assessments that may be required~~. **If Investigator consider that additional safety assessment is needed, additional blood samples may be required.**

Rationale: According to investigator's suggestion to specify the times and volume of blood sample collecting.

Change 9

Section 7.2.2 Vital Signs, Paragraph 2 and Section 7.2.6 Electrocardiogram (ECG), Paragraph 2

PREVIOUS TEXT

At each timepoint, assessments should be performed after the subject has been resting in a supine/semi-supine position for at least 10 minutes.

ECGs should be performed after the subject has rested in a supine/semi-supine position for at least 10 minutes. Additional ECGs may be taken at the discretion of the investigator as needed based on signs or symptoms.

REVISED TEXT

Vital sign measurements will include systolic and diastolic blood pressure and pulse rate.

At each timepoint, assessments should be performed after the subject has been resting in a supine/semi-supine position for at least ~~10~~ **5** minutes.

ECGs should be performed after the subject has rested in a supine/semi-supine position for at least ~~10~~ **5** minutes. Additional ECGs may be taken at the discretion of the investigator as needed based on signs or symptoms.

Rationale: According to investigator's suggestion to shorten the interval between the examination of vital signs and ECG.

Change 10

Section 7.2.7 Clinical Laboratory Assessments

PREVIOUS TEXT

Clinical Chemistry

BUN	Potassium	AST	Total bilirubin
Creatinine	Chloride	ALT	Direct bilirubin
Glucose, fasting	GGT	Albumin	Albumin
Sodium	Calcium	Alkaline phosphatase	Total Protein
Serum β-hCG(women of child bearing potential)			

Routine Urinalysis

Specific gravity
pH, glucose, protein, blood, bilirubin, creatinine and ketones by dipstick
Microscopic examination (if blood or protein is abnormal)

Other screening tests

HIV
Serum hCG test
Hepatitis B (HBsAg)
Hepatitis C (Hep C antibody)
FSH and estradiol (as needed in women of non-child bearing potential only)
Alcohol and drug screen (to include at minimum: amphetamines, barbiturates, cocaine, opiates, cannabinoids and benzodiazepines).

REVISED TEXT

Clinical Chemistry

BUN	Potassium	AST	Total bilirubin
Creatinine	Chloride	ALT	Direct bilirubin
Glucose, fasting	GGT	~~Albumin~~ **<u>Total Protein</u>**	Albumin
Sodium	Calcium	Alkaline phosphatase	
~~Serum β-hCG(women of child bearing potential)~~			

<u>NOTE: Details of Liver Chemistry Follow-up Procedures are given in Section 13.</u>

Routine Urinalysis

Specific gravity
pH, glucose, protein, blood, bilirubin~~, creatinine~~ and ketones by dipstick
Microscopic examination (if blood or protein is abnormal)

Other screening tests

HIV
**Syphilis (syphilis antibody)**
Serum hCG test **at screening, urine hCG test prior to dosing (day -1)**
Hepatitis B (HBsAg)
Hepatitis C (Hep C antibody)
FSH and estradiol (as needed in women of non-child bearing potential only)
Alcohol and drug screen (to include at minimum: amphetamines, barbiturates, cocaine, opiates, cannabinoids and benzodiazepines).

Rationale: Delete the creatinine in the routine urinalysis as it cannot be tested by dipstick method. Typo correction and according to investigator's suggestion to add syphilis test and specify pregnancy test at screening.

Change 11

Section 7.4.1 Blood Sample Collection

PREVIOUS TEXT

Blood samples for pharmacokinetic analysis of GSK2371746 will be collected at the time points indicated in Section 4.5 Time and Events Table. The actual date and time of each blood sample collection will be recorded. The timing of PK samples may be altered and/or PK samples may be obtained at additional time points to ensure thorough PK monitoring.

Details of PK blood sample collection (including volume to be collected), processing, storage and shipping procedures are provided in the Study Procedures Manual (SPM).

REVISED TEXT

Blood samples **(approximately 3.5 mL)** for pharmacokinetic analysis of GSK2371746 will be collected at the time points indicated in Section 4.5 Time and Events Table. The actual date and time of each blood sample collection will be recorded. The timing of PK samples may be altered and/or PK samples may be obtained at additional time points to ensure thorough PK monitoring.

Details of PK blood sample ~~collection (including volume to be collected),~~ processing, storage and shipping procedures are provided in the Study Procedures Manual (SPM).

Rationale: Specify the volume of blood samples collection for PK analysis according to investigator's suggestion.

Change 12

Section 7.4.2 Sample Analysis

PREVIOUS TEXT

Serum analysis will be performed under the management of PPD, Virginia, USA. Concentrations of GSK2371746 will be determined using the currently approved analytical methodology. Raw data will be stored in the archives at PPD. Further details on sample processing are included in the Study Procedures Manual.

REVISED TEXT

~~Serum analysis will be performed under the management of PPD, Virginia, USA.~~ Concentration~~s~~ **determination** of GSK2371746 **in serum** will be **performed by Wuxi AppTec** ~~determined~~ using ~~the currently approved~~ **a** **validated bioanalytical** methodology. Raw data will be stored in the **Good Laboratory Practices Archives** at ~~PPD~~ **Wuxi AppTec for five years before sponsor transfer request**. ~~Further details on sample processing are included in the Study Procedures Manual.~~ **Further details on methodology and sample analysis results will be included in the method validation and sample analysis reports.**

Rationale: PK sample analysis will be performed by the laboratory in China.

Change 13

Section 7.5.1 Confirmed Biomarkers/Pharmacodynamic Markers

PREVIOUS TEXT

Serum samples will be assayed for s-CTX1. Refer to the Study Procedures Manual for detailed instructions on collection and processing of these samples. No other biomarkers will be collected or analyzed during this study.

REVISED TEXT

s-CTX1 is selected as pharmacodynamic marker in this study. Blood samples (approximately 3.5 mL) should be taken in the morning after an overnight fast. Serum samples will be assayed for s-CTX1 **by the electrochemiluminescence immunosassay.** Refer to the Study Procedures Manual for detailed instructions on collection and processing of these samples. No other biomarkers will be collected or analyzed during this study.

Rationale: Specify the volume of blood sampling and detecting method of s-CTX1 according to investigator’s suggestion.

Change 14

Section 7.6 Denosumab Antibody (Seroreactivity) Assay

PREVIOUS TEXT

Blood samples for Denosumab antibody (binding and neutralizing) analysis will be collected at the time points indicated in Section 4.5, Time and Events Table. Refer to the Study Procedures Manual for detailed instructions on collection and processing of these samples.

REVISED TEXT

Blood samples **(approximately 7 mL)** for Denosumab antibody (binding and neutralizing) analysis will be collected at the time points indicated in Section 4.5, Time and Events Table. **Denosumab antibody analysis will be assayed by Frontage. If the serum sample is positive for an antibody to denosumab, serum sample(s) will be exported to the laboratory in Amgen for examination in a confirmatory assay.** Refer to the Study Procedures Manual for detailed instructions on collection and processing of these samples.

Rationale: Specify the volume of blood sampling and detecting methods of denosumab antibody according to investigator's suggestion.

Change 15

Section 10.3.1 Subject Withdrawal from Study, the last paragraph

PREVIOUS TEXT

If a subject is withdrawn from the study, perform all of the procedures described in the Time and Events Table for the last clinical visit (unless they have withdrawn informed consent).

REVISED TEXT

If a subject is withdrawn from the study, perform all of the procedures described in the Time and Events Table for the ~~last clinical~~ **early withdrawal** visit (unless they have withdrawn informed consent).

Rationale: Clarify the early withdrawal visit instead of last clinical visit to avoid confusion.

Change 16

Section 10.5 Screen and Baseline Failures

PREVIOUS TEXT

Data for screen and baseline failures will be collected in source documentation at the site but will not be transmitted to GSK.

REVISED TEXT

Data for screen and baseline failures will be collected in source documentation at the site ~~but will not be transmitted to GSK~~. **Minimal screen failure information will be collected on CRF, including Demography, Screen Failure details, Eligibility Criteria and Serious Adverse Events.**

Rationale: According to the GSK new policy, it is required to collect the screen failure data.

Change 17

Section 11.1 Description of Investigational Product, Paragraph 3

PREVIOUS TEXT

Investigators will provide calcium and vitamin D supplements to all subjects randomized into the study. The recommended administration of these supplements is at least 1000 mg calcium (daily) and at least 400 IU vitamin D daily for the duration of the study.

REVISED TEXT

Investigators will provide calcium and vitamin D supplements to all subjects randomized into the study. The recommended administration of these supplements is at least ~~1000~~ **600** mg calcium (daily) and at least 400 IU vitamin D but <1000 IU daily for the duration of the study.

Rationale: Correct the calcium supplementation dose to ensure it is aligned with that in denosumb China postmenopausal osteoporosis phase III study.

Change 18

Section 11.3 Blinding, Paragraph 1

PREVIOUS TEXT

This will be a single-blind study. The subjects and site staff will be blinded.

The investigator or treating physician may unblind a subject's treatment assignment only in the case of an emergency, when knowledge of the study treatment is essential for the appropriate clinical management or welfare of the subject. Whenever possible, the investigator must first discuss options with the GSK Medical Monitor or appropriate GSK study personnel before unblinding the subject's treatment assignment. If this is impractical, the investigator must notify GSK as soon as possible, but without revealing the treatment assignment of the unblinded subject, unless that information is important for the safety of subjects currently in the study. The date and reason for the unblinding must be recorded in the appropriate data collection tool.

REVISED TEXT

This will be a single-blind study. The subjects ~~and site staff~~ will be blinded, **GSK and site staff will be unblinded.**

~~The investigator or treating physician may unblind a subject's treatment assignment only in the case of an emergency, when knowledge of the study treatment is essential for the appropriate clinical management or welfare of the subject. Whenever possible, the investigator must first discuss options with the GSK Medical Monitor or appropriate GSK study personnel before unblinding the subject's treatment assignment. If this is impractical, the investigator must notify GSK as soon as possible, but without revealing the treatment assignment of the unblinded subject, unless that information is important for the safety of subjects currently in the study. The date and reason for the unblinding must be recorded in the appropriate data collection tool.~~

Rationale: Clarify the blinding is to subjects only. Delete the remnant description of unblinding because the site staff and sponsor are not blinded.

Change 19

Section 12 ADVERSE EVENTS (AE) AND SERIOUS ADVERSE EVENTS (SAE)

PREVIOUS TEXT

No previous text.

REVISED TEXT

Add

12.4: Atypical Femoral Fracture

Femoral fracture adverse events will be reviewed by an independent adjudication committee for suspicion of being an atypical femoral fracture (AFF). AFF are defined as subtrochanteric or proximal diaphyseal fractures that occur with little to no trauma and are characterized by specific radiographic findings.

Screening will consist of regular review of AE and SAE reports, and by using a predefined list of related Medical Dictionary for Regulatory Activities (MedDRA) preferred terms. For identified cases of possible AFF, GSK will request the investigating site provide all available source documents surrounding that event and completed an AFF adjudication questionnaire. An independent adjudication panel of blinded AFF experts will determine if diagnostic criteria are met. If an event is adjudicated positively for AFF the investigator will be notified of the adjudication decision; and the events will be upgraded to an SAE if not already reported as such, and reported to the regulatory agencies and study investigators in an expedited manner.

Rationale: Add atypical femoral fracture information into AE and SAE.

Change 20

Section 14.9 Adjudication Committee

PREVIOUS TEXT

14.9 Adjudication Committee

If at any time there are safety concerns, the ONJAC will communicate the concerns to GSK.

REVISED TEXT

14.9 Osteonecrosis of the Jaw Adjudication Committee

~~If at any time there are safety concerns, the ONJAC will communicate the concerns to GSK.~~

Rationale: According to GCSP’s comments.

Change 21

PREVIOUS TEXT

No previous text.

REVISED TEXT

Add

14.10. Atypical Femoral Fracture Adjudication Committee (AFFAC)

Cases of atypical femoral fracture have been reported in the osteoporosis literature in association with long-term bisphosphonate use. Some case series have reported a possible association between atypical femoral fracture and long-term alendronate therapy, with low bone turnover as the suggested etiology [Lenart, 2008; Odvina, 2005; Odvina, 2010], while others have not [Abrahamsen, 2009]. Atypical femoral fracture has been confirmed in an ongoing clinical trial 20060289 with denosumab. All events reported as atypical femoral fractures, or those coded to pre-specified terms potentially indicative of atypical femoral fractures, will be reviewed by an independent adjudication committee.

Rationale: Add atypical femoral fracture adjudication committee information.

AMENDMENT 1

Where the Amendment Applies

This amendment applies to all sites.

Summary of Amendment Changes with Rationale

The primary intent of this amendment is to change the study subjects from healthy adult volunteers in Hong Kong and Taiwan to healthy adults in China, based on the purpose of this study is to meet regulatory requirements for the registration of denosumab in China. The number of subjects is halved as subjects come from one region, and this sample size could still meet the requirement of China regulatory guidance on pharmacokinetics study. Additional changes are listed as below.

- Update the unit in authors list and sponsor signatory page.
- Change the responsible person on the sponsor/medical monitor information page and sponsor registered address because the study is transferred from central team to China team.
- Provide agency indentifying number.
- Update information of clinical studies in Safety profile is based on the new data in updated version of Investigator's Brochure.
- Simplify study rationale.
- Clarify ±2 days for Visit Window from Week 3 to follow-up in Time and Events Table.
- Change the age range from "between 20 and 65" to "18 and 65" as this change could expand the inclusion population and much more close to the requirement of China regulatory guidance on pharmacokinetics study.
- Clarify using serum β-hCG pregnancy test for women.
- Clarify the caffeine and alcohol consumption in Section 8.3.
- Clarify the period of corticosteroids administration in Section 9.1.
- Provide updated version of Clinical Investigator's Brochure as reference.

List of Specific Changes

<u>Change 1</u>

Title:

PREVIOUS TEXT

A Randomized, Single-blind, Parallel-group, Placebo-controlled, Single-dose Study to Evaluate the Safety, Tolerability, Pharmacokinetics, and Pharmacodynamics of Denosumab Administered Subcutaneously to Healthy Adults in Hong Kong and Taiwan

REVISED TEXT

A Randomized, Single-blind, Parallel-group, Placebo-controlled, Single-dose Study to Evaluate the Safety, Tolerability, Pharmacokinetics, and Pharmacodynamics of Denosumab Administered Subcutaneously to Healthy Adults in ~~Hong Kong and Taiwan~~ China

Description:

PREVIOUS TEXT

Denosumab is currently under development for the treatment of bone loss indications. This study represents the first administration of denosumab in Hong Kong and Taiwan populations. The primary purpose of the study is to assess the safety and tolerability of denosumab in healthy adult volunteers in Hong Kong and Taiwan. The study will also characterize the pharmacokinetic and pharmacodynamic properties of denosumab after single, subcutaneous administration.

REVISED TEXT

Denosumab is currently under development for the treatment of bone loss indications. This study represents the first administration of denosumab in ~~Hong Kong and Taiwan populations~~ China. The primary purpose of the study is to assess the safety and tolerability of denosumab in healthy adult **Chinese** volunteers ~~in Hong Kong and Taiwan~~. The study will also characterize the pharmacokinetic and pharmacodynamic properties of denosumab after single, subcutaneous administration.

Rationale: Change study subjects from healthy adult volunteers in Hong Kong and Taiwan to healthy adults in China, which is based on the purpose of this study to meet regulatory requirements for the registration of denosumab in China.

Change 2

Author:

PREVIOUS TEXT

[redacted] CVM MDC

[redacted] MDC Clinical MGU

REVISED TEXT

[redacted] ~~CVM MDC~~ **MPC TA Unit**

[redacted] ~~MDC Clinical MGU~~ **MPC TA Unit**

SPONSOR SIGNATORY:

PREVIOUS TEXT

[redacted] MD
Medicine Development Leader
Biopharm R&D

REVISED TEXT

[redacted] MD
Medicine Development Leader
~~Biopharm R&D~~ Metabolic Pathway & Cardiovascular

Change 3

SPONSOR/MEDICAL MONITOR INFORMATION PAGE:
PREVIOUS TEXT

Medical Monitor and Sponsor Contact Information:

**Role**	Name	Day Time Phone Number	After-hours Phone/Cell/ Pager Number	Fax Number	GSK Address
Primary Medical Monitor	[redacted] MD	[redacted]	[redacted]	[redacted]	2301 Renaissance Boulevard King of Prussia, PA 19406
Secondary Medical Monitor	[redacted] MD	[redacted]	[redacted]	[redacted]	2301 Renaissance Boulevard King of Prussia, PA 19406
SAE fax number				[redacted]	

Sponsor Registered Address:

GlaxoSmithKline
Five Moore Drive
PO Box 13398
Research Triangle Park, NC 27709
USA

Regulatory Agency Identifying Number(s): NA

REVISED TEXT

Medical Monitor and Sponsor Contact Information:

**Role**	Name	Day Time Phone Number	After-hours Phone/Cell/ Pager Number	Fax Number	GSK Address
Primary Medical Monitor	[redacted] MD	[redacted]	[redacted]	[redacted]	No.1 building, 917 Halei Road Zhangjiang Hi-tech Park, Pudong, shanghai
Secondary Medical Monitor	[redacted] MD	[redacted]	[redacted]	[redacted]	No.1 building, 917 Halei Road Zhangjiang Hi-tech Park, Pudong, shanghai
SAE fax number				[redacted]	

Sponsor Registered Address:

GlaxoSmithKline
2301 Renaissance Boulevard
Building #510
Post Office Box 61540
King of Prussia, Pennsylvania 19406
Telephone number: [redacted]

Regulatory Agency Identifying Number(s): 2013L02177

Rationale: Change the responsible person on the sponsor/medical monitor information page and sponsor registered address because the study is transferred from central team to China team. Regulatory agency identifying number is provided according to the clinical trial approval form.

Change 4

ABBREVIATIONS:

PREVIOUS TEXT

ALT	Alanine aminotransferase (SGPT)
AST	Aspartate aminotransferase (SGOT)
…	…
SGOT	Serum glutamic oxaloacetic transaminase

SGPT	Serum glutamic pyruvic transaminase

REVISED TEXT

ALT	Alanine aminotransferase ~~(SGPT)~~
AST	Aspartate aminotransferase ~~(SGOT)~~
…	…
~~SGOT~~	~~Serum glutamic oxaloacetic transaminase~~
~~SGPT~~	~~Serum glutamic pyruvic transaminase~~

Rationale: Avoid duplicated term.

Change 5

Trademark Information:

PREVIOUS TEXT

Trademarks of the GlaxoSmithKline group of companies	Trademarks not owned by the GlaxoSmithKline group of companies
NONE	Amgen
	Chiron RIBA
	InForm
	NONMEM
	PPD
	SAS
	WinNonlin

REVISED TEXT

Trademarks of the GlaxoSmithKline group of companies	Trademarks not owned by the GlaxoSmithKline group of companies
NONE	~~Amgen~~ **WinNonlin**
	Chiron RIBA
	~~InForm~~ **InForm**
	~~NONMEN~~ **Amgen**
	PPD
	SAS
	~~WinNonlin~~

Change 6

Section 1.1.3.1 Safety Profile, Paragraph 2

PREVIOUS TEXT

A comprehensive clinical program is currently evaluating denosumab as a potential treatment for postmenopausal osteoporosis (PMO), male osteoporosis, bone loss due to hormone-ablation therapy in subjects with cancer, inhibition of structural damage in subjects with rheumatoid arthritis, prevention of bone metastases in at risk populations, prevention of skeletal-related events in subjects with advanced malignancies involving bone (including multiple myeloma), treatment of multiple myeloma, treatment of giant cell tumor, and treatment of hypercalcemia of malignancy. As of 23 December 2009, approximately 21,500 subjects have enrolled in clinical studies and have received at least 1 dose of investigational product (i.e., denosumab [approximately 14,000 subjects], matching placebo, or active control). Cumulative doses up to 1080 mg over 6 months have been evaluated in the advanced cancer setting without evidence of dose-limiting toxicity. Repeated fixed subcutaneous (SC) doses of up to 210 mg Q6M have been studied for up to 24 months in postmenopausal women with low bone mineral density (BMD). Additionally, SC doses of up to 180 mg 4- or 12-weekly (Q4W or Q12W) have been studied for up to 26 weeks in subjects with cancer-related bone metastases, and SC doses of 60 and 180 mg Q6M have been studied for up to 12 months in subjects with rheumatoid arthritis. Key safety findings from these studies of denosumab support the following conclusions:

REVISED TEXT

A comprehensive clinical program is currently evaluating denosumab as a potential treatment for postmenopausal osteoporosis (PMO), male osteoporosis, bone loss due to hormone-ablation therapy in subjects with cancer, inhibition of structural damage in subjects with rheumatoid arthritis, prevention of bone metastases in at risk populations, prevention of skeletal-related events in subjects with advanced malignancies involving bone (including multiple myeloma), treatment of multiple myeloma, treatment of giant cell tumor, and treatment of hypercalcemia of malignancy. As of ~~23 December 2009~~ **May 2013**, approximately ~~21,500~~ **32,800** subjects have enrolled in clinical studies and have received at least 1 dose of investigational product (i.e., denosumab ~~[approximately 14,000 subjects]~~, matching placebo, or active control). Cumulative doses up to 1080 mg over 6 months have been evaluated in the advanced cancer setting without evidence of dose-limiting toxicity. Repeated fixed subcutaneous (SC) doses of up to 210 mg Q6M have been studied for up to 24 months in postmenopausal women with low bone mineral density (BMD). Additionally, SC doses of up to 180 mg 4- or 12-weekly (Q4W or Q12W) have been studied for up to 26 weeks in subjects with cancer-related bone metastases, and SC doses of 60 and 180 mg Q6M have been studied for up to 12 months in subjects with rheumatoid arthritis. Key safety findings from these studies of denosumab support the following conclusions:

Rationale: Update information of clinical studies in Safety profile is based on the new data in updated version of Investigator’s Brochure.

Change 7

Section 1.1.5 Pharmacokinetic Profile, Paragraph 3

PREVIOUS TEXT

For additional information on denosumab, please refer to the Clinical Investigator's Brochure [Denosumab (AMG 162) Investigator's Brochure, 2010].

REVISED TEXT

For additional information on denosumab, please refer to the Clinical Investigator's Brochure [Denosumab (AMG 162) Investigator's Brochure, ~~2010~~ **Edition 2.0, 23 August 2013**].

Rationale: Provide updated version of Clinical Investigator's Brochure as reference.

Change 8

Section 1.2 Study Rationale

PREVIOUS TEXT

The purpose of this study is to provide safety, tolerability, pharmacokinetic and pharmacodynamic data for denosumab in healthy volunteers resident in Hong Kong and Taiwan in order to support planned Phase III bridging studies in these ethnic populations to meet regulatory requirements for the registration of denosumab for the treatment of bone loss indications (i.e., postmenopausal osteoporosis (PMO), hormone ablation therapy (HALT) in men with prostate cancer and women with breast cancer) and oncology indication (i.e., skeletal-related events in patients with advanced cancer and bone metastases; SRE).

[Lau, 1996] studied the body composition and bone mineral density of Chinese women with vertebral fracture. They compared the body composition and bone mineral density measurements (BMD) in Chinese women with vertebral fracture and normal controls. A total of 400 community dwelling Chinese women aged 70–79 years old were studied. Vertebral height ratios were calculated from lateral thoracic and lumbar spine X-rays and subjects were classified into definite cases (n = 122), doubtful cases (n = 138) and normal controls (n = 140). Bone mineral density and body composition measurements were made by dual X-ray densitometry. The height, fat mass, lean mass, and BMD at all sites were significantly lower in patients with definite fracture than normal controls. Nevertheless, BMD at the hip was more predictive of vertebral fracture than BMD at the spine, the odds ratio in the lowest quartile of hip BMD being 3.8 (95% C.I. = 1.3 to 10.9).

REVISED TEXT

The purpose of this study is to provide safety, tolerability, pharmacokinetic and pharmacodynamic data for denosumab in healthy **Chinese** volunteers ~~resident in Hong Kong and Taiwan~~ in order to ~~support planned Phase III bridging studies in these ethnic~~

~~populations to~~ meet regulatory requirements for the registration of denosumab ~~for the treatment of bone loss indications (i.e., postmenopausal osteoporosis (PMO), hormone ablation therapy (HALT) in men with prostate cancer and women with breast cancer) and oncology indication (i.e., skeletal-related events in patients with advanced cancer and bone metastases; SRE)~~.

~~[Lau, 1996] studied the body composition and bone mineral density of Chinese women with vertebral fracture. They compared the body composition and bone mineral density measurements (BMD) in Chinese women with vertebral fracture and normal controls. A total of 400 community dwelling Chinese women aged 70–79 years old were studied. Vertebral height ratios were calculated from lateral thoracic and lumbar spine X-rays and subjects were classified into definite cases (n = 122), doubtful cases (n = 138) and normal controls (n = 140). Bone mineral density and body composition measurements were made by dual X-ray densitometry. The height, fat mass, lean mass, and BMD at all sites were significantly lower in patients with definite fracture than normal controls. Nevertheless, BMD at the hip was more predictive of vertebral fracture than BMD at the spine, the odds ratio in the lowest quartile of hip BMD being 3.8 (95% C.I. = 1.3 to 10.9).~~

Rationale: Revise the region of study subjects and simplify the study rationale.

Change 9

Section 1.4 Summary of Risk Management, Paragraph 1

PREVIOUS TEXT

Denosumab has been generally well tolerated in all clinical studies conducted to date. This study represents the first trial involving subjects in Hong Kong and Taiwan.

REVISED TEXT

Denosumab has been generally well tolerated in all clinical studies conducted to date. This study represents the first trial involving subjects in ~~Hong Kong and Taiwan~~ **China**.

Rationale: Revise the region of study subjects from Hong Kong and Taiwan to China.

Change 10

Section 1.4 Summary of Risk Management, Paragraph 5

PREVIOUS TEXT

Detailed information on safety is provided in the Investigator’s Brochure [Denosumab (AMG 162) Investigator’s Brochure, 2010].

REVISED TEXT

Detailed information on safety is provided in the Investigator's Brochure [Denosumab (AMG 162) Investigator's Brochure, ~~2010~~ **Edition 2.0, 23 August 2013**].

Rationale: Provide updated version of Clinical Investigator's Brochure as reference.

Change 11

Section 2.1 Primary Objective

PREVIOUS TEXT

- To assess the safety and tolerability of denosumab in healthy, adult subjects in Hong Kong and Taiwan

REVISED TEXT

- To assess the safety and tolerability of denosumab in healthy, adult **Chinese** subjects ~~in Hong Kong and Taiwan~~

Section 2.2 Secondary Objective

PREVIOUS TEXT

- To characterize the pharmacokinetic and pharmacodynamic properties of denosumab after single SC administration in healthy, adult subjects in Hong Kong and Taiwan

REVISED TEXT

- To characterize the pharmacokinetic and pharmacodynamic properties of denosumab after single SC administration in healthy, adult **Chinese** subjects ~~in Hong Kong and Taiwan~~

Rationale: Revise the region of study subjects from Hong Kong and Taiwan to China.

Change 12

Section 4.1 Study Design/Schematic

PREVIOUS TEXT

This is a randomized, parallel-group, single-blind, placebo-controlled study which will be conducted in a total of approximately 128 healthy volunteer subjects to assess the safety and tolerability of denosumab. Subjects will be randomized to denosumab 60 mg, denosumab 120 mg and placebo in a ratio of 3:3:2. Approximately 48 subjects (24 in Hong Kong and 24 in Taiwan) will be randomized to each active treatment group and 32

(16 in Hong Kong and 16 in Taiwan) to placebo in order to complete approximately 20 subjects in Hong Kong for each active treatment group and 20 subjects in Taiwan for each active treatment group.

The randomization schedule will be stratified by population (subjects in Hong Kong and subjects in Taiwan) and gender to achieve the 1:1 ratio between male and female within each population. Reasonable diligence will be used to enrol about an equal number of males as females.

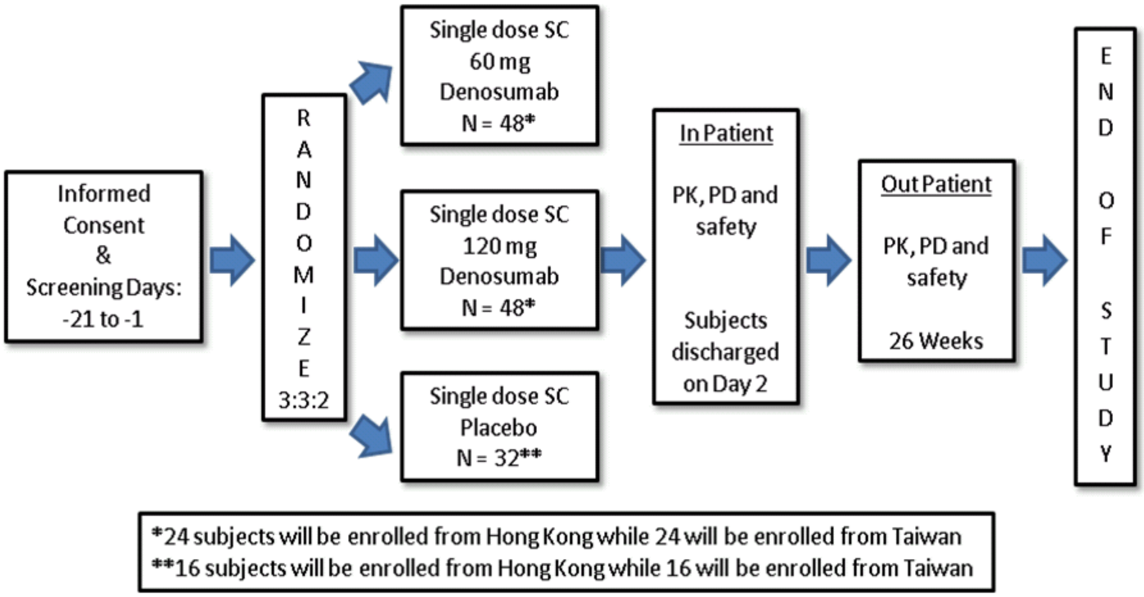

Figure 2 Study Design Schematic

REVISED TEXT

This is a randomized, parallel-group, single-blind, placebo-controlled study which will be conducted in a total of approximately ~~128~~ **64** healthy volunteer subjects to assess the safety and tolerability of denosumab. Subjects will be randomized to denosumab 60 mg, denosumab 120 mg ~~and~~ **or** placebo in a ratio of 3:3:2. Approximately ~~48~~ **24** subjects ~~(24 in Hong Kong and 24 in Taiwan)~~ will be randomized to each active treatment group and ~~32~~**16** ~~(16 in Hong Kong and 16 in Taiwan)~~ to placebo in order to complete approximately 20 subjects ~~in Hong Kong~~ for each active treatment ~~group and 20 subjects in Taiwan for each active treatment group~~.

~~The randomization schedule will be stratified by population (subjects in Hong Kong and subjects in Taiwan) and gender to achieve the 1:1 ratio between male and female within each population.~~ Reasonable diligence will be used to enrol about an equal number of males as females.

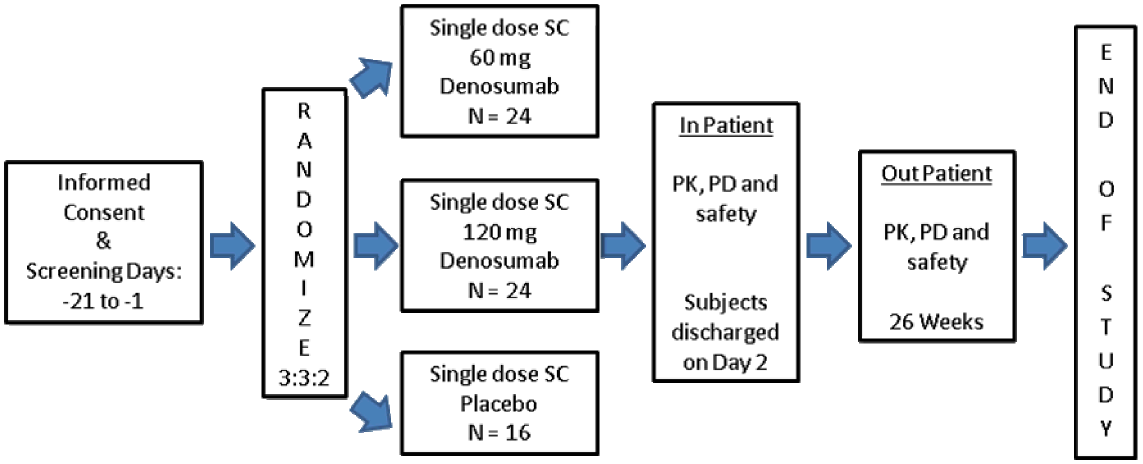

Figure 3 Study Design Schematic

Rationale: Number of subjects is halved as the region of subjects is changed from two to one.

Change 13

Section 4.2 Discussion of Design, Paragraph 1

PREVIOUS TEXT

This study employs a randomized, single-blind, parallel, placebo-controlled design. The design employed for this study is identical to the design of the previous Phase 1 studies conducted by Amgen [Amgen Protocol Number 20050146 and Amgen Protocol Number 20060446].

REVISED TEXT

This study employs a randomized, single-blind, parallel, placebo-controlled design. The design employed for this study is identical to the design of the previous Phase 1 studies conducted by Amgen ~~[Amgen Protocol Number 20050146 and~~ (Amgen Protocol Number 20060446~~]~~).

Change 14

Section 4.5 Time and Events Table

PREVIOUS TEXT

Day:	Screening	-1	1	2	3	4	5	6	8	11											Follow-up (via Phone)
**Week:**											3	4	5	7	9	11	13	15	17	19	26
**Visit Window** (relative to Day 1)	-21 to -1 days																				
**Admission to Unit**		X																			
**Informed Consent**	X																				
**Inclusion/Exclusion**	X																				
**Demographics**	X																				
**Physical Examination**	X																			X	
**Medical history**	X																				
**12-lead ECG**	X[1]																			X	
**Vital signs(BP and HR)**	X	X	X[2]	X	X	X	X	X	X	X	X	X	X	X	X	X	X	X	X	X	
**Height**	X																				
**Weight**	X																			X	
**Urine drug/alc. screen**	X	X																			
**β-hCG pregnancy test (women)**	X	X																			
**HIV, Hep B and Hep C**	X																				
**Hema/Chem/Urinalysis**	X								X[3]				X				X			X	
**AE assessment**	X[4]	X[4]	X	X	X	X	X	X	X	X	X	X	X	X	X	X	X	X	X	X	X
**Pregnancy assessment phone call**																					X
**Conmed Review**			X	X	X	X	X	X	X	X	X	X	X	X	X	X	X	X	X	X	
**PK blood sample**			X[5]	X[5]	X[5]	X	X	X	X	X	X	X	X	X	X	X	X	X	X	X	
**PD (CTX1) sample**			X[2]	X	X	X	X	X	X	X	X	X	X	X	X	X	X	X	X	X	
**Antibody sample**			X[2]									X			X					X	

**Day:**	Screening	-1	1	2	3	4	5	6	8	11											Follow-up (via Phone)
**Week:**											3	4	5	7	9	11	13	15	17	19	26
**Visit Window** (relative to Day 1)	-21 to -1 days																				
**Dosing**			X																		
**Discharge**				X																	
**Outpatient visit**	X				X	X	X	X	X	X	X	X	X	X	X	X	X	X	X	X	

REVISED TEXT

**Day:**	Screening	-1	1	2	3	4	5	6	8	11											Follow-up (via Phone)
**Week:**											3	4	5	7	9	11	13	15	17	19	26
**Visit Window** (relative to Day 1)	-21 to -1 days	**NA**									**±2 days**										
**Admission to Unit**		X																			
**Informed Consent**	X																				
**Inclusion/Exclusion**	X																				
**Demographics**	X																				
**Physical Examination**	X																			X	
**Medical history**	X																				
**12-lead ECG**	X[1]																			X	
**Vital signs(BP and HR)**	X	X	X[2]	X	X	X	X	X	X	X	X	X	X	X	X	X	X	X	X	X	
**Height**	X																				
**Weight**	X																			X	
**Urine drug/alc. screen**	X	X																			
**Serum β-hCG pregnancy test (women)**	X	X																			
**HIV, Hep B and Hep C**	X																				
**Hema/Chem/Urinalysis**	X								X[3]				X				X			X	
**AE assessment**	X[4]	X[4]	X	X	X	X	X	X	X	X	X	X	X	X	X	X	X	X	X	X	X

Day:	Screening	-1	1	2	3	4	5	6	8	11											Follow-up (via Phone)
**Week:**											3	4	5	7	9	11	13	15	17	19	26
**Visit Window** (relative to Day 1)	-21 to -1 days	**<u>NA</u>**									**<u>±2 days</u>**										
**Pregnancy assessment phone call**																					X
**Conmed Review**			X	X	X	X	X	X	X	X	X	X	X	X	X	X	X	X	X	X	
**PK blood sample**			X<br>5	X<br>5	X<br>5	X	X	X	X	X	X	X	X	X	X	X	X	X	X	X	
**PD (CTX1) sample**			X<br>2	X	X	X	X	X	X	X	X	X	X	X	X	X	X	X	X	X	
**Antibody sample**			X<br>2									X			X					X	
**Dosing**			X																		
**Discharge**				X																	
**Outpatient visit**	X				X	X	X	X	X	X	X	X	X	X	X	X	X	X	X	X	

Rationale: Clarify ±2 days for Visit Window from Week 3 to follow-up and clarify using serum β-hCG pregnancy test for women.

<u>Change 15</u>

Section 5.1 Number of Subjects

PREVIOUS TEXT

Approximately 64 subjects will be enrolled in Hong Kong such that approximately 20 subjects per active dose group and 14 subjects in the placebo group complete dosing and critical assessments. Similarly, approximately 64 subjects will be enrolled in Taiwan such that approximately 20 subjects per active dose group and 14 subjects in the placebo group complete dosing and critical assessments.

If subjects prematurely discontinue the study, additional subjects may be enrolled as replacement subjects and assigned to the same treatment at the discretion of the Sponsor and in consultation with the investigator.

REVISED TEXT

Approximately 64 subjects will be enrolled ~~in Hong Kong~~ such that approximately 20 subjects per active dose group and 14 subjects in the placebo group complete dosing and critical assessments. ~~Similarly, approximately 64 subjects will be enrolled in Taiwan such that approximately 20 subjects per active dose group and 14 subjects in the placebo group complete dosing and critical assessments.~~

~~If subjects prematurely discontinue the study, additional subjects may be enrolled as replacement subjects and assigned to the same treatment at the discretion of the Sponsor and in consultation with the investigator.~~

Rationale: Number of subjects is halved as the region of subjects is changed from two to one. Replacement for prematurely discontinued subjects is not applicable in the study.

Change 16

Section 5.2.1 Inclusion Criteria

PREVIOUS TEXT

1. Subjects in Hong Kong – defined as being resident in Hong Kong and of Chinese ancestry.

   OR

   Subjects in Taiwan – defined as being resident in Taiwan and of Chinese ancestry.

   …

4. Subjects in Hong Kong – male or female between 18 and 65 years of age, inclusive, from date of birth, at the time of signing the informed consent.

OR

   Subjects in Taiwan – male or female between 20 and 65 years of age, inclusive, from date of birth, at the time of signing the informed consent.

REVISED TEXT

1. Resident in China and of Chinese ancestry.

   …

4. Male or female between **18** and 65 years of age, inclusive, from date of birth, at the time of signing the informed consent

Rationale: Revise the region of study subjects from Hong Kong and Taiwan to China. Change the age range from “between 20 and 65” to “18 and 65” as it could expand the inclusion population and be closer to the requirement of China regulatory guidance on pharmacokinetics study.

Change 17

Section 5.2.2 Exclusion Criteria

PREVIOUS TEXT

15. Pregnant females as determined by positive serum or urine hCG test at screening or prior to dosing.

REVISED TEXT

15. Pregnant females as determined by positive serum ~~or urine~~ hCG test at screening or prior to dosing **(visit -1)**.

Rationale: Clarify using serum hCG pregnancy test for women.

Change 18

Section 5.2.3 Other Eligibility Criteria Considerations

PREVIOUS TEXT

To assess any potential impact on subject eligibility with regard to safety, the investigator must refer to the following document(s) for detailed information regarding warnings, precautions, contraindications, adverse events, and other significant data pertaining to the investigational product(s) being used in this study: [Denosumab (AMG 162) Investigator's Brochure, 2010].

REVISED TEXT

To assess any potential impact on subject eligibility with regard to safety, the investigator must refer to the following document(s) for detailed information regarding warnings, precautions, contraindications, adverse events, and other significant data pertaining to the investigational product(s) being used in this study: [Denosumab (AMG 162) Investigator's Brochure, ~~2010~~ **Edition 2.0, 23 August 2013**].

Rationale: Provide updated version of Clinical Investigator's Brochure as reference.

Change 19

Section 6.2.1 Sample Size Assumptions, Paragraph 1

PREVIOUS TEXT

A sufficient number of subjects will be enrolled so that approximately 108 subjects (20 subjects in Hong Kong and 20 subjects in Taiwan for 60 mg group, 20 subjects in Hong Kong and 20 subjects in Taiwan for 120 mg group, and 14 subjects in Hong Kong and 14 subjects in Taiwan for placebo group) complete the study. Sample size is based in part on feasibility. However, some justification is provided below.

REVISED TEXT

~~A sufficient number of~~ **Approximately 64** subjects will be enrolled so that approximately ~~108~~ **54**subjects (20 subjects ~~in Hong Kong and 20 subjects in Taiwan~~ for 60 mg group, 20 subjects ~~in Hong Kong and 20 subjects in Taiwan~~ for 120 mg group, and 14 subjects ~~in Hong Kong and 14 subjects in Taiwan~~ for placebo group) complete the study. Sample size is based in part on feasibility. However, some justification is provided below.

Rationale: Number of subjects is halved as the region of subjects is changed from two to one.

Change 20

Section 6.3.2.1 Safety Analyses

PREVIOUS TEXT

Safety data will be presented by population (subjects in Hong Kong and subjects in Taiwan) and may be combined also in tabular and/or graphical format and summarized descriptively according to GSK's Integrated Data Standards Library (IDSL) standards.

REVISED TEXT

Safety data will be presented ~~by population (subjects in Hong Kong and subjects in Taiwan) and may be combined also~~ in tabular and/or graphical format and summarized descriptively according to GSK's Integrated Data Standards Library (IDSL) standards.

Section 6.3.2.2 Pharmacokinetic Analyses, Paragraph 4

PREVIOUS TEXT

Pharmacokinetic parameters will be summarized by population (subjects in Hong Kong and subjects in Taiwan) and treatment group. Mean, Median, Min, Max, SD, GeoMean, log SD, CV, 90% CI will be provided; Individual and mean PK concentration-time curves will be provided. Population combined summary may be provided.

REVISED TEXT

Pharmacokinetic parameters will be summarized by ~~population (subjects in Hong Kong and subjects in Taiwan) and~~ treatment group. Mean, Median, Min, Max, SD, GeoMean, log SD, CV, 90% CI will be provided; Individual and mean PK concentration-time curves will be provided. ~~Population combined summary may be provided.~~

Section 6.3.2.4 Pharmacodynamic/Biomarker Analyses, Paragraph 3

PREVIOUS TEXT

PD parameters (serum CTX1 AUEC and Imax) will be summarized by population (subjects in Hong Kong and subjects in Taiwan) and treatment group; Mean, Median, Min, Max, SD, GeoMean, log SD, CV, 90% CI will be provided; Individual and mean CTX1 inhibition curves will be provided. Population combined summary may be provided.

REVISED TEXT

PD parameters (serum CTX1 AUEC and Imax) will be summarized by ~~population (subjects in Hong Kong and subjects in Taiwan) and~~ treatment group; Mean, Median, Min, Max, SD, GeoMean, log SD, CV, 90% CI will be provided; Individual and mean CTX1 inhibition curves will be provided~~. Population combined summary may be provided.~~

Rationale: Change the region of subjects from Hong Kong and Taiwan to China.

Change 21

Section 7.2.7 Clinical Laboratory Assessments

PREVIOUS TEXT

Clinical Chemistry

BUN	Potassium	AST (SGOT)	Total bilirubin
Creatinine	Chloride	ALT (SGPT)	Direct bilirubin
Glucose, fasting	GGT	Albumin	Albumin
Sodium	Calcium	Alkaline phosphatase	Total Protein
Serum β-hCG (women of child-bearing potential)			

Other screening tests

HIV
Hepatitis B (HBsAg)
Hepatitis C (Hep C antibody -- if second generation Hepatitis C antibody positive, a hepatitis C antibody Chiron RIBA immunoblot assay should be reflexively performed **on the same sample** to confirm the result)
FSH and estradiol (as needed in women of non-child bearing potential only)
Alcohol and drug screen (to include at minimum: amphetamines, barbiturates, cocaine, opiates, cannabinoids and benzodiazepines).

REVISED TEXT

Clinical Chemistry

BUN	Potassium	AST ~~(SGOT)~~	Total bilirubin
Creatinine	Chloride	ALT ~~(SGPT)~~	Direct bilirubin
Glucose, fasting	GGT	Albumin	Albumin
Sodium	Calcium	Alkaline phosphatase	Total Protein
Serum β-hCG (women of child-bearing potential)			

Other screening tests

HIV
**Serum hCG test**
Hepatitis B (HBsAg)
Hepatitis C (Hep C antibody ~~-- if second generation Hepatitis C antibody positive, a hepatitis C antibody Chiron RIBA immunoblot assay should be reflexively performed **on the same sample** to confirm the result~~)
FSH and estradiol (as needed in women of non-child bearing potential only)
Alcohol and drug screen (to include at minimum: amphetamines, barbiturates, cocaine, opiates, cannabinoids and benzodiazepines).

Rationale: To avoid the duplicated term and clarify using serum hCG pregnancy test for women. HCV RIBA isn't available anywhere in China; Quest reflexes to HCV DNA as confirmatory test.

Change 22

Section 8.3 Caffeine, Alcohol, and Tobacco, Paragraph 1

PREVIOUS TEXT

Subjects will abstain from ingesting caffeine- or xanthine-containing products (e.g. coffee, tea, cola drinks, oolong tea, green tea, herbal teas, and chocolate) for 24 hours prior to the start of dosing until collection of the final pharmacokinetic and or pharmacodynamic sample during the in-house portion of the study.

REVISED TEXT

~~Subjects will abstain~~ **Caffeine consumption during the study should not change substantially** from ~~ingesting caffeine- or xanthine-containing products (e.g. coffee, tea, cola drinks, oolong tea, green tea, herbal teas, and chocolate)~~ **pre-study levels** for 24 hours prior to the start of dosing until collection of the final pharmacokinetic and or pharmacodynamic sample during the in-house portion of the study.

Alcohol consumption should not exceed an average weekly intake of 14 drinks/week for men or 7 drinks/week for women. One drink is equivalent to (12 g alcohol) = 150 ml (5 ounces) of table wine or 360 ml (12 ounces) of beer or 45 ml (1.5 ounces) of 80 proof distilled spirits.

Rationale: Caffeine and alcohol consumption could be acceptable in this study.

Change 23

Section 9.1 Permitted Medications, Paragraph 2

PREVIOUS TEXT

Corticosteroids (inhaled or topical) administered more than 2 weeks prior to enrollment will be permitted.

REVISED TEXT

Corticosteroids (inhaled or topical) administered more than 2 weeks prior to ~~enrollment~~ **enrolment and continuing throughout the study** will be permitted.

Rationale: Clarify the period of corticosteroids administration.

Change 24

REFERENCES

PREVIOUS TEXT

Denosumab (AMG 162) Investigator's Brochure, Edition 9.0, 05 February 2010

REVISED TEXT

Denosumab (AMG 162) Investigator's Brochure, Edition ~~9.0~~ **2.0**, ~~05 February 2010~~ **23 August 2013**

Rationale: Provide updated version of Clinical Investigator's Brochure as reference.
